# Multi-Tissue Computational Modeling Analyzes Pathophysiology of Type 2 Diabetes in MKR Mice

**DOI:** 10.1371/journal.pone.0102319

**Published:** 2014-07-16

**Authors:** Amit Kumar, Thomas Harrelson, Nathan E. Lewis, Emily J. Gallagher, Derek LeRoith, Joseph Shiloach, Michael J. Betenbaugh

**Affiliations:** 1 Department of Chemical and Biomolecular Engineering, Johns Hopkins University, Baltimore, Maryland, United States of America; 2 Biotechnology Core Laboratory, National Institute of Diabetes and Digestive and Kidney Diseases, National Institute of Health, Bethesda, Maryland, United States of America; 3 Department of Biology, Brigham Young University, Provo, Utah, United States of America; 4 Division of Endocrinology, Diabetes and Bone Disease and the Diabetes, Obesity, Metabolism Institute, Icahn School of Medicine at Sinai, New York, New York, United States of America; CRCHUM-Montreal Diabetes Research Center, Canada

## Abstract

Computational models using metabolic reconstructions for *in silico* simulation of metabolic disorders such as type 2 diabetes mellitus (T2DM) can provide a better understanding of disease pathophysiology and avoid high experimentation costs. There is a limited amount of computational work, using metabolic reconstructions, performed in this field for the better understanding of T2DM. In this study, a new algorithm for generating tissue-specific metabolic models is presented, along with the resulting multi-confidence level (MCL) multi-tissue model. The effect of T2DM on liver, muscle, and fat in MKR mice was first studied by microarray analysis and subsequently the changes in gene expression of frank T2DM MKR mice versus healthy mice were applied to the multi-tissue model to test the effect. Using the first multi-tissue genome-scale model of all metabolic pathways in T2DM, we found out that branched-chain amino acids' degradation and fatty acids oxidation pathway is downregulated in T2DM MKR mice. Microarray data showed low expression of genes in MKR mice versus healthy mice in the degradation of branched-chain amino acids and fatty-acid oxidation pathways. In addition, the flux balance analysis using the MCL multi-tissue model showed that the degradation pathways of branched-chain amino acid and fatty acid oxidation were significantly downregulated in MKR mice versus healthy mice. Validation of the model was performed using data derived from the literature regarding T2DM. Microarray data was used in conjunction with the model to predict fluxes of various other metabolic pathways in the T2DM mouse model and alterations in a number of pathways were detected. The Type 2 Diabetes MCL multi-tissue model may explain the high level of branched-chain amino acids and free fatty acids in plasma of Type 2 Diabetic subjects from a metabolic fluxes perspective.

## Introduction

Type 2 Diabetes Mellitus (T2DM), the most common form of Diabetes in America, is becoming a global pandemic with the greatest increase in cases in many developing countries. The pathophysiology of T2DM primarily involves defects in three organ systems– liver, peripheral target tissues (skeletal muscle and fat), and pancreatic β-cells [1]. Insulin resistance in the peripheral target tissues, primarily skeletal muscle, is considered the primary reason for insulin resistance in T2DM [2].

In the patients with T2DM, withdrawal of insulin treatment has been shown to be associated with increased levels of branched-chain amino acids (BCAAs) in the plasma [3]. Moreover, metabolite profiling from the plasma of T2DM patients [4] revealed BCAAs as the key-biomarkers during the progression of T2DM. It is shown that the concentrations of BCAAs in plasma, liver, and skeletal muscle are higher in T2DM conditions such as in the Zucker diabetic rat [5]. Another study performed on hyperglycemic/T2DM Finnish males revealed high plasma level of BCAAs [6] too. Additionally, it has been shown that high levels of BCAAs in plasma of T2DM subjects are associated with conditions of insulin-resistance [7]–[12].

It is also known that elevated free fatty acid (FFA) levels in plasma is linked to T2DM in patients [13]. One of the studies [14] on the effect of high plasma FFA levels, pointed out the contribution of high FFA levels in plasma on the impaired insulin response of the T2DM subjects.

In contrast to the wealth of knowledge available for the concentrations of circulating BCAAs and FFAs in T2DM patients, the actual mechanisms leading to these changes at the metabolic and genetic levels are less understood. With the emergence of systems biology tools associated with high-throughput data, it is now feasible to create *in silico* genome scale metabolic reconstruction models to study the causes of various metabolic disorders [15]. After completion of a global human metabolic network [16], Recon1, constraint-based modeling became feasible to study metabolic disorders *in silico*. Accounting for more than 3000 human metabolic reactions, Recon1 provides a firm basis for studying human metabolism and metabolic disorders such as cancer, diabetes, obesity, and inherited gene and enzyme deficiencies [15].

Following Recon1 coming into existence, several tissue-specific models have also been generated [17]–[22]. Recon1 in itself is not sufficient for modeling specific tissues as different tissues exhibit different physiological and hence metabolic behaviors. Increased efforts are creating multi-tissue metabolic models to study the pathophysiology of human metabolic disorders [23].

Insulin resistance leading to Type 2 Diabetes Mellitus (T2DM) is regulated by more than one tissue system requiring analysis at multi-tissue level. Major tissues involved in T2DM are skeletal muscle, liver, adipose, pancreas, brain, and gastrointestinal tract [24]. As far as the level of metabolites is concerned, skeletal muscle, liver, and adipose tissues are the major role players in secreting these metabolites into the blood by various biochemical pathways. Metabolite concentration levels (such as amino acids levels) from T2DM subjects' blood is readily available in literature, allowing comparison of an *in silico* model to T2DM phenotype. The current study introduces a comprehensive multi-tissue-specific model to study interdependence of hepatocytes, mycoytes, and adipocytes in the T2DM condition. Table 1 shows a summary of some different models including the model for the current study (Kumar et al.) [17], [18], [20], [25], [26]. Each model provides specific advantages and limitation for specific applications. Models can vary in size and scope and also tissue distribution. For example, while some models are specific for certain tissues, the current Kumar model expands the scope to include three tissues. Moreover, microarray data used in our study contextualizes the reconstruction according to the three different tissues (liver, WAT, and skeletal muscle). Furthermore, we used microarray data for three different tissues from the same animals. While we recognize that the current approach incorporates this data from a different organism (mice) into a model based on human metabolism, the similarity in physiological responses across species makes such an approach reasonable in the absence of fully validated models and data sets for each species. Most of the studies involving T2DM and insulin resistance utilize data from insulin-resistant or diabetic animal models, such as Zucker fatty (ZF) and Zucker diabetic fatty rats (ZDF)[27], high-fat-fed mice[28], muscle IGF-1 receptor–lysine–arginine (MKR) mice[29], [30], and lep/lep mice[31]. A common feature of these animal models is that all models have manifested insulin resistance and often exhibit islet dysfunction as occurs in the early stages of type 2 diabetes in humans. It is, in our opinion, valid to use MKR mice and Zucker diabetic fatty rat data for making predictions on humans' T2DM phenotypes. To validate the model, its capability to predict expected phenotypes from known genotypes was tested. For this purpose, a publically available comprehensive database for genes and genetic phenotypes, the Online Mendelian Inheritance in Man (OMIM) database was used. This was followed by a comparison between constraint-based simulation results and the levels of plasma amino acid in the Zucker diabetic rat after gene expression data for the three tissues from the diabetic MKR mouse compared to normal control mice [32].

**Table 1:** A comparison of different models.

	Kumar	Bordbar	Jerby	Gille	Mardinoglu	Recon1
**Intracellular Reaction**	2202	518	1056	1081	6160	2180
**Genes**	1496	931	-	-	1809	1496
**Unique Metabolites**	610	413	729	777	2497	1509
**Compartments**	4	4	6	6	8	7

A comparison of intracellular reaction, number of distinct genes, number of unique metabolites, and total compartments in different metabolic reconstructions.

## Results

### A.1 Multi-confidence level (MCL) multi-tissue model

**Figure 1:**
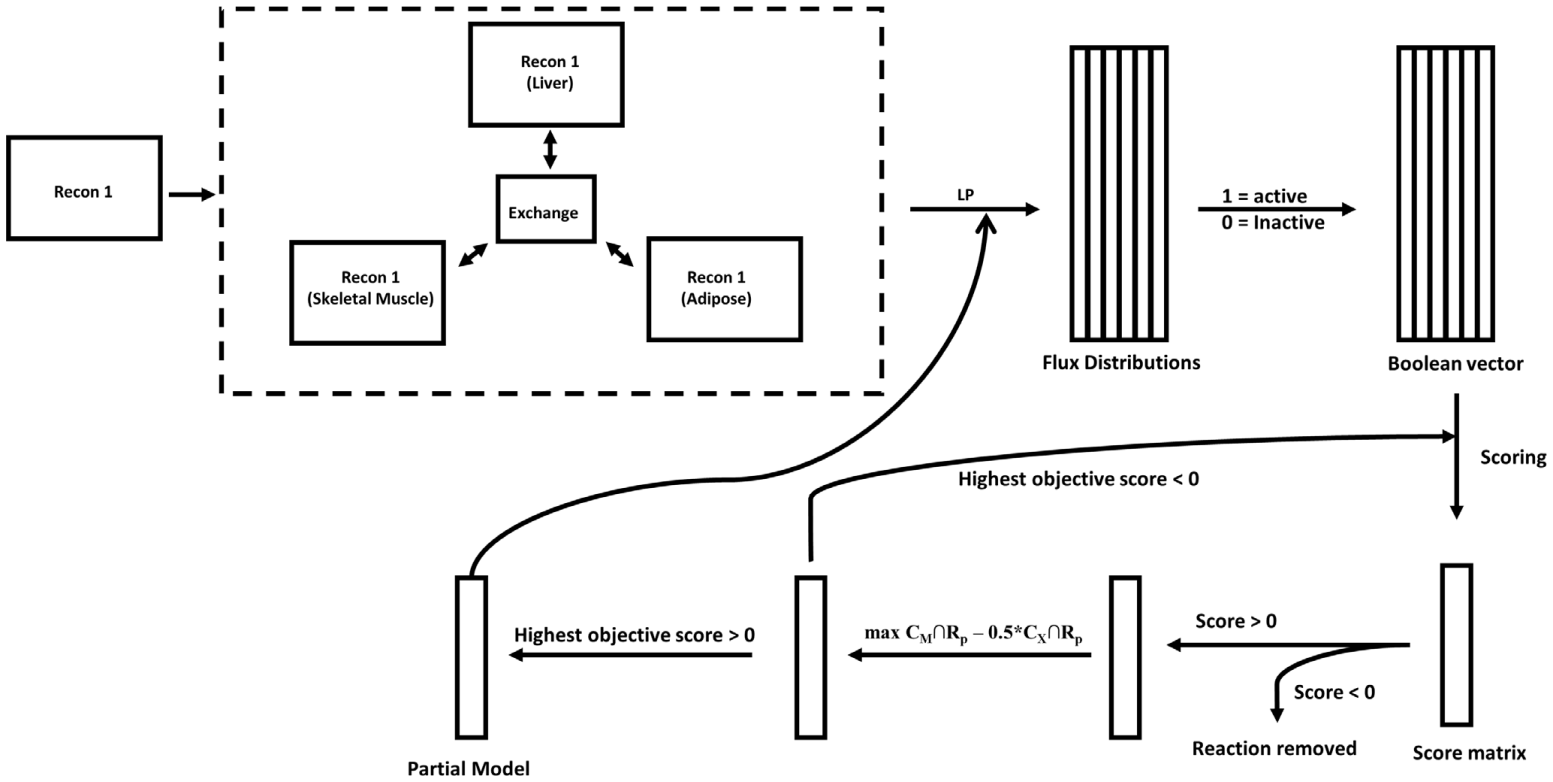
Multi-tissue model building workflow. Recon1, downloaded from BiGG database, is the basis of building this multi-tissue model for liver, skeletal muscle, and adipose tissues. From the Recon1 individual compartment models, the algorithm first loads a randomized flux distribution matrix representing randomization of linear programming (LP) problems. Then a Boolean vector describing activity (1 =  active, 0 =  inactive) of each reaction is created. Then scoring for each column of the flux distribution is done. If the objective score is greater than 0, corresponding reaction is added in the construction. This process is repeated until highest objective score is greater than 0, implying objective function is at its maximum. This generates a partial model. Then the whole process is repeated again based on the partial model, until there is no change in model size between one iteration to another iteration.

Before generating the model, an algorithm was applied to generate a list of high confidence, medium confidence, and low confidence reactions, based on the source of a particular reaction. The high confidence list comprised reactions from published literature and was not altered by the algorithm; the medium confidence list comprised of reactions from online databases such as HPRD, etc.; and the low confidence list comprised of all remaining reactions that weren't present in high and medium confidence reaction lists, as shown in Figure 1 (detailed in Materials and Methods). The final high confidence reaction list (C_h_) consists of 1110 reactions and 1593 metabolites. The final medium confidence reaction list (C_m_) consists of 1099 reactions and 1643 metabolites. The final low confidence reaction list (C_x_) consists of 4433 reactions and 3679 metabolites. The algorithm required six iterations before completing to give multi-confidence level (MCL) multi-tissue model with 4704 reactions and 3131 metabolites. Completion is indicated by the fact that the objective function for the whole model is at a maximum for a particular set of flux distributions and no more reactions can be added in subsequent iterations (detailed in Materials and Methods).

### A.2 Validation using Online Mendelian Inheritance in Man (OMIM) Database

**Figure 2:**
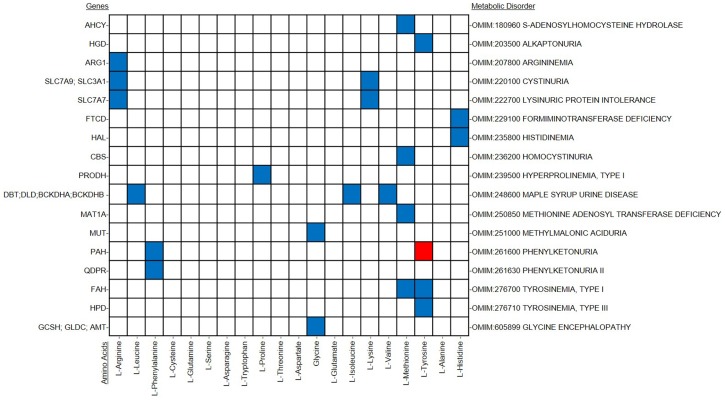
OMIM data used for model validation. Blue and red squares depict an increase and decrease in concentration, respectively. White squares represent unchanged concentration levels.

**Figure 3:**
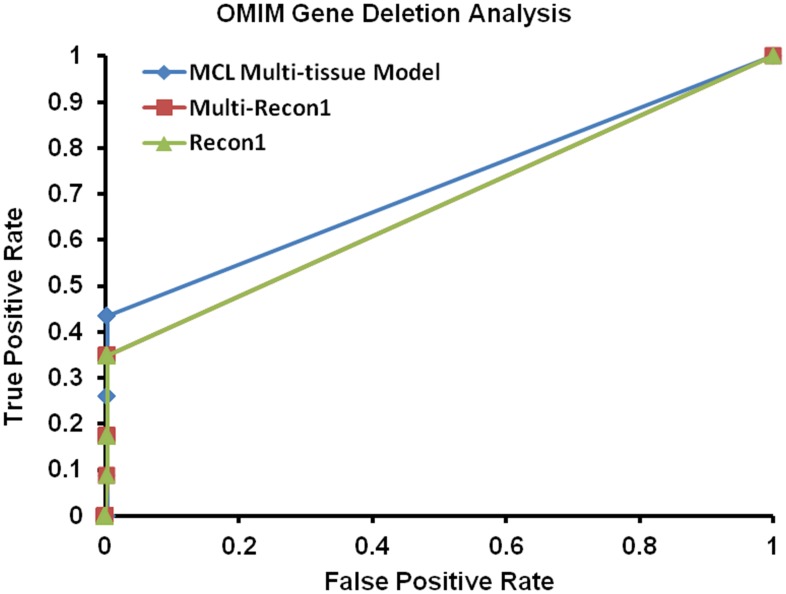
ROC plot for validation using OMIM data. ROC plot comparing different models using gene-deletion study. This figure demonstrates the true positive and false positive rates at various threshold values.

- From the OMIM database [33], 17 disorders were chosen and characterized with regard to increases or decreases in metabolite level in the blood as shown in Figure 2 [22]. Each of the disorders involves changes in the amino acid levels. The phenotype of each of these disorders is outlined in Figure 2 above. Each disorder has a set of associated genes, as listed in Figure 2, which can be used to map to reactions *in silico*. S-Adenosylhomocysteine hydrolase is associated with AHCY gene deficiency [34]. Alkaptonuria is associated with HGD gene deficiency [35]. Argininemia is associated with mutation in ARG1 gene [36]. Cystinuria is associated with mutations in SLC3A1 and SLC7A9 genes [37]. Lysinuric protein intolerance is associated with mutation in SLC7A7 gene [38]. Formiminotransferace deficiency is associated with mutation in FTCD gene [39]. Histidinemia is associated with mutation in HAL gene [40]. Homocystinuria is associated with mutation in CBS gene [41]. Hyperprolinemia is associated with mutation in PRODH gene [42]. Maple syrup urine disease is associated with mutation in DBT, BCKDHB, and BCKDHA genes [43]. Methionine adenosyltransferase deficiency is caused by mutation in MAT1A gene [44]. Methylmalonic aciduria is caused by mutation in MUT gene [45]. Phenylketonuria is caused by mutation in PAH gene [46]. Hyperphenylalaninemia is associated with mutation in QDPR [47]. Tyrosinemia, Type I is caused by mutation in FAH gene [48]. Tyrosinemia, type III is caused by mutation in HPD gene [49]. Glycine encephalopathy is associated with mutation in AMT, GLDC, and GCSH genes [50]. In this validation, the reactions associated with these genes were removed and simulation of the *in silico* model was run to predict the phenotype of the disease associated with removing that particular gene(s). The predicted phenotype was then compared to the actual phenotype from the OMIM database. Exchange reactions tell about the concentration and the exchange flux balances indicate the increase or decrease in concentration of a metabolite in the model in the blood/extracellular compartment. This analysis was performed on the MCL multi-tissue model, Recon1, and the multi-tissue version of Recon1 (modeling the three tissues: adipose, liver, and skeletal muscle). The results from simulations of Recon1 and the multi-tissue version of Recon1 served as benchmarks to compare to the simulations of MCL multi-tissue model, as shown in receiver-operator curves (ROC) in Figure 3. ROC curves portray the trade-off between true positive rate (TPR) and false positive rate (FPR) predictor of a model as the decision threshold of a parameter is varied.

Recon1 and the multi-tissue version of Recon1 have an identical curve which is understandable as a multi-tissue version of Recon1 is just a combination of Recon1 models of the three tissues and the external exchange compartment; however, it is necessary to test the differences between the two because the number of type III cycles can increase in the multi-tissue version of Recon1. A type III cycle is one of three types of extreme pathways that can exist in a reaction network (type I, type II, and type III) and could cause the behavior of the multi-tissue version to be different than the single tissue version of Recon1. Figure 3 illustrates that the MCL multi-tissue model reaches higher true positive rates as compared to Recon1. Also, all data points other than the last one at (1,1) are clustered in an area with a very low false positive rate. This indicates that all models evaluated in this experiment performed as expected. The area under the curve (AUC) provides a quantitative measure of the performance of each model. The AUC of the MCL multi-tissue model is 0.7151, and the AUC of Multi – Recon1 and Recon1 is 0.6719, and AUC of a random selector (not shown) is approximately 0.3866.

### A.3 Validation using Type 2 Diabetes Gene Expression Change

Subsequent validation analysis involved the application of microarray data from MKR T2DM mice to the aforementioned *in silico* models. The microarray data of the two sets was compared and genes that had statistically significant (with p-value <0.05) fold changes were tabulated. These differentially expressed genes were then mapped to Recon1 and the MCL multi-tissue model to determine sets of upregulated and downregulated reactions. The bounds of these reactions were changed according to the procedure defined elsewhere (Materials and Methods). The resulting differences in the exchange reactions flux bounds in both models were compared with amino acid data for the Zucker diabetic fatty rat from the literature [5], as shown in Table 2. With respect to the reference model with no change; any positive value corresponds to upregulated and any negative value corresponds to downregulated.

**Table 2:** Simplified depiction of the validation results.

Amino acid	Zucker diabetic fatty rat	Ex-Recon1	Ex-MCL multi-tissue Model	Trans-Recon1	Trans-MCL multi-tissue Model
Arginine	↓	↑	-	-	↓
Leucine	↑	-	-	↑	-
Phenylalanine	-	↑	-	-	-
Cysteine	-	-	-	↓	-
Glutamine	↓	-	↑	↑	-
Serine	↓	↑	-	↑	-
Asparagine	↓	-	-	↑	-
Tryptophan	↓	-	-	-	↓
Proline	-	↓	-	-	-
Threonine	↓	-	-	↑	-
Aspartate	-	↑	↑	-	-
Glycine	↓	-	-	↓	-
Glutamate	↑	-	-	↓	-
Isoleucine	↑	-	↑	-	↑
Lysine	↓	-	-	-	↑
Valine	↑	-	-	↑	↑
Methionine	↓	↑	↑	-	↑
Tyrosine	↓	-	↓	↓	↓
Alanine	-	↓	↑	-	-
Histidine	↓	↑	↓	↓	↑

The upward arrow (↑) depicts higher amino acid level in Zucker diabetic fatty rat versus healthy rat. The downward arrow (↓) depicts lower amino acid level in Zucker diabetic fatty rat versus healthy rat. (-) depicts no difference in the amino acid level between the two types of rats.

The difference between the two pairs (Recon 1 and MCL multi-tissue model) of columns in Table 2 represent two different analyses in which reactions were used in calculating increases and decreases in plasma concentration of the corresponding amino acids. The columns labeled “Ex-Recon1” and “Ex-MCL Multi-tissue Model” represent analyses in which the exchange reactions were used solely to determine the increases and decreases in concentration of amino acids in the blood/plasma. The columns labeled “Trans-Recon1” and “Trans-MCL Multi-tissue Model” represent analyses in which transport reactions were used to determine the increases and decreases in different pathways' fluxes. In this instance, a transport reaction is defined as a reaction that moves a particular amino acid from the cytosol to the extracellular/blood compartment. An exchange reaction is a special type of reaction that only exists in these types of computational models and represent flow of metabolites across a system boundary [51].

**Figure 4:**
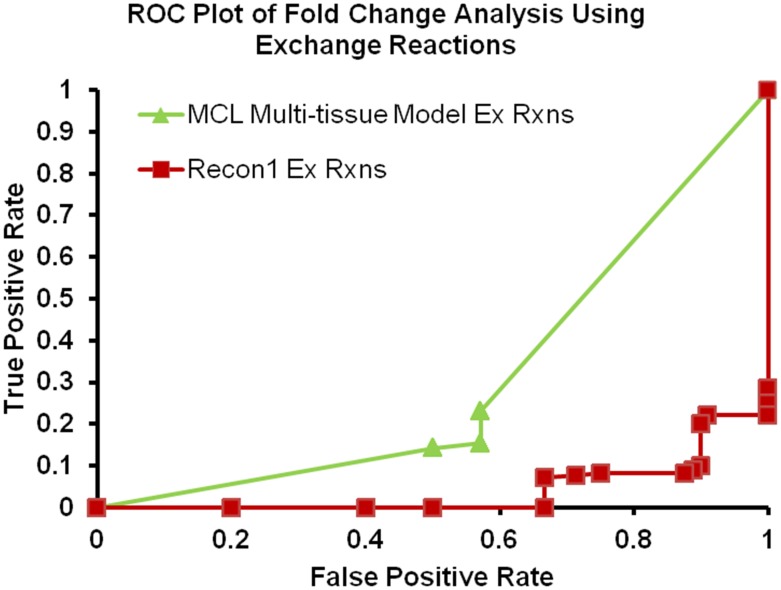
ROC plot for exchange flux changes using Zucker fatty rat data. ROC plot of the results generated by comparing the exchange flux changes determined by applying diabetic and wild-type gene expression data to the MCL multi-tissue model and Recon1 to available literature data on amino acid concentration changes in the blood/plasma.

**Figure 5:**
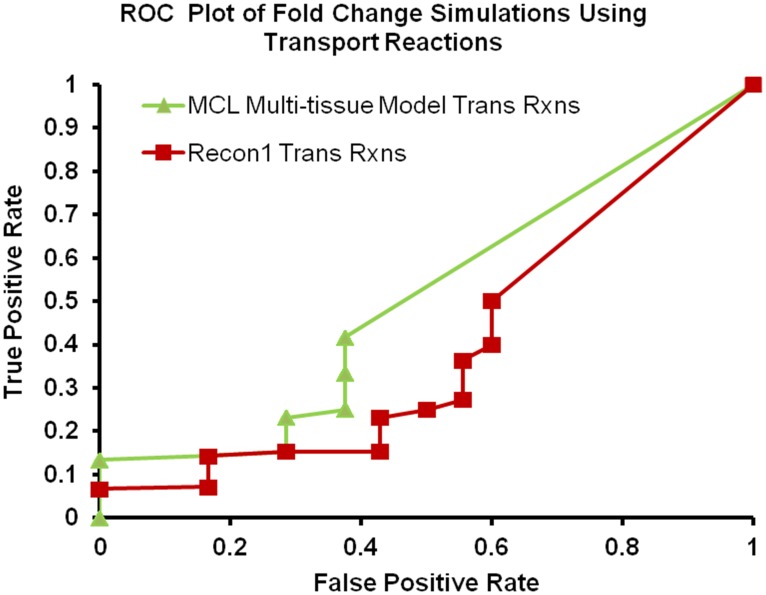
ROC plot for transport reaction flux changes using Zucker fatty rat data. ROC plot of the results generated by the flux changes in transport reactions determined by applying T2DM and wild-type gene expression data to the MCL multi-tissue model and Recon1and then comparing the results to available literature data on amino acid concentration changes in the plasma of T2DM Zucker diabetic fatty rat.

The first two rows in Table 3 represent the analyses using exchange reactions, and as expected the MCL multi-tissue model outperforms Recon1 in every category (a lower FPR is better). The last two rows in Table 3 represent the analyses using the transport reactions. Table 3 shows that the MCL multi-tissue model significantly outperformed Recon1 when using exchange reactions, but the results appear to be much closer when using transport reactions. The MCL multi-tissue model outperformed Recon1 in every category except recall (recall is same as true positive rate). ROC curves for the differences in flux bounds of the exchange reactions and transport reactions are shown in Figure 4 and Figure 5 respectively.

**Table 3:** Summarized results of validation based on T2DM gene expression change.

Model	TP	FP	TN	FN	Precision	Recall	TNR	FPR	Accuracy
Ex-Recon1	0	8	1	11	0.00	0.00	0.11	0.89	0.05
Ex-MCL multi-tissue Model	3	4	3	10	0.43	0.23	0.43	0.57	0.30
Trans-Recon1	5	6	4	5	0.45	0.50	0.40	0.60	0.45
Trans-MCL multi-tissue Model	5	3	5	7	0.63	0.42	0.63	0.38	0.50

TP stands for True Positive, FP stands for False Positive, TN stands for True Negative, FN stands for False Negative, TNR stands for True Negative Rate, and FPR stands for False Positive Rate. As seen from the Accuracy column, MCL multi-tissue model outperforms Recon1 is both exchange and transport reactions.

These ROC curves clearly demonstrate the differences between Recon1 and MCL multi-tissue model by providing the clear differences in the area under the curve (AUC) between the two models. In Figure 4, the MCL multi-tissue model outperformed Recon1, which isn't surprising given the results in Table 3. In the figures 4 and 5, there are more points in Recon1 because of the fact that there are greater number of reactions in Recon1 as compared to the multi-tissue model and therefore downregulated genes were mapped to more reactions. The AUC of the fold change simulations of MCL multi-tissue model using Transport reactions in Figure 4 is 0.3100; the AUC of Recon1 in Figure 4 is 0.0412 and the AUC of random selector (not shown) is 0.3866. The AUC's of the MCL multi-tissue model using Exchange reactions and Recon1 in Figure 5 are 0.5048 and 0.3998, respectively, which means that both Recon1 and the MCL multi-tissue model are better than a random selector for transport reactions. Thus, the MCL multi-tissue model is consistently above Recon1 for both transport and exchange reactions and exceeds the random selector curve for transport reactions but not for exchange reactions. The random selector curve is based on a set of reactions generated by random permutation from the entire set of reactions.

Recon1 was highly inaccurate for transport reactions at each threshold, which demonstrates the difficulty with predicting increases or decreases of exchange from gene expression fold changes. The fact that the MCL multi-tissue model displays an AUC that is roughly 7.5 times greater than the AUC produced by Recon1 provides further validation for the MCL multi-tissue model in these analyses, at least compared to other systems available.

### A.4 MCL multi-tissue model's application on MKR mice microarray data

After the two extensive validations outlined in sections 2.1 and 2.2, the model was used to study physiological behaviors during T2DM condition – higher plasma concentration of branched-chain amino acids (BCAAs) and free fatty acids (FFAs) in T2DM subjects. Branched-chain amino acid metabolism has been studied in T2DM condition previously [10], [52], [53], but none of the groups have determined metabolic fluxes through relevant pathways, as described in this study using a robust computational model. Past studies [18], [17] have focused on a single tissue (such as liver) for investigating metabolic disorders. However, this doesn't give a complete picture of the metabolic disorder. In another study [20] the investigators studied T2DM on fat, liver, and muscle tissues using an *in silico* model which was less comprehensive in terms of the number of metabolic reactions used in the one presented in this study. In order to use the MCL multi-tissue model for predicting physiological changes in fully T2DM MKR mice as compared to healthy mice, gene expression data was first generated for liver, skeletal muscle, and fat tissues for healthy and diabetic mice. Statistical tests on gene expression data suggested that several genes were differentially regulated between MKR and healthy mice. The statistical test – Fisher's exact test, providing significance of the association of the experimental gene expression values and the Ingenuity Pathway Analysis canonical pathways, was done using the Ingenuity software (www.ingenuity.com). From gene expression fold change analysis in the three tissues, it was found that for BCAA degradation and FA oxidation pathways, some of the genes are upregulated and some are downregulated in liver, while the same were predominantly downregulated in the T2DM MKR mice's fat and muscle tissues when compared to their healthy euglycemic littermates, as shown in Table 4.

**Table 4:** Microarray results for BCAA and FA degradation pathways.

Tissue Pathway	Valine Degradation (%)	Isoleucine Degradation (%)	Leucine Degradation (%)	Tyrosine Degradation (%)	Phenylalanine Degradation (%)	Fatty-acid Oxidation (%)
Fat	81↓/0↑	79↓/0↑	55↓/0↑	30↓/30↑	10↓/10↑	50↓/10↑
Liver	15↓/0↑	10↓/0↑	No change	0↓/20↑	0↓/20↑	5↓/5↑
Skeletal Muscle	35↓/0↑	30↓/0↑	0↓/15↑	50↓/0↑	50↓/20↑	40↓/0↑

Percentage of genes downregulated (↓)/upregulated (↑) in BCAA degradation and FA oxidation pathways in the three tissues.

Liver tissues’ FA degradation pathways’ genes did not provide a clear explanation of high concentration.

**Figure 6:**
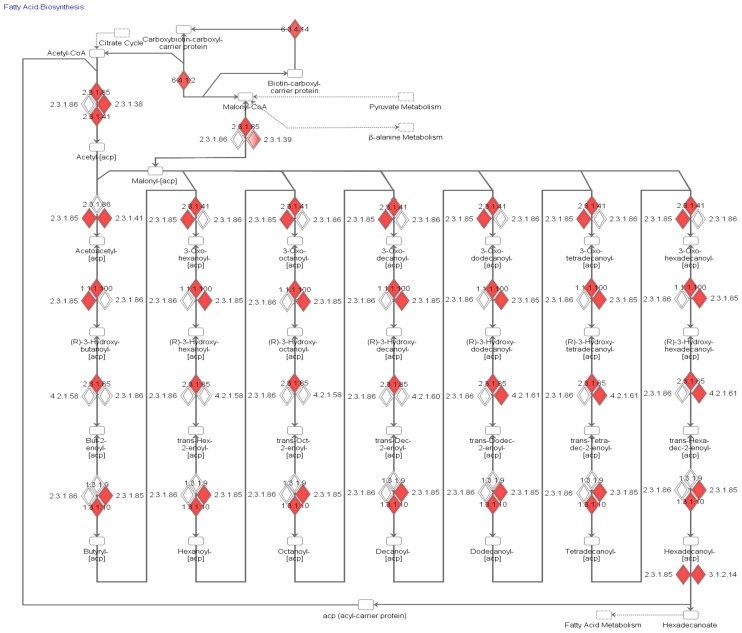
Canonical biosynthesis pathway of fatty acids in liver using microarray data. Mapping of gene expression for liver tissues on biosynthesis of fatty acids, using Ingenuity Pathway Analysis software. Red color shows upregulated gene expression in MKR mice versus Healthy mice.

The liver tissues' FA degradation pathway genes did not provide a clear explanation of high concentration of FFAs in the plasma of T2DM subjects. However, the biosynthesis pathway from Ingenuity Pathway Analysis (Figure 6) for fatty acids in the liver tissues of MKR mice does provide an explanation of the differences in FFA levels in healthy and diseased subjects' plasma. There is a clear increase in the gene expression patterns for biosynthesis of fatty acids in liver and a decrease in fatty acid oxidation in skeletal muscle and fat tissues, thereby accumulating more free fatty acids in the plasma of T2DM subjects.

In order to perform systems level analysis of the gene expression data, to achieve a better understanding of BCAA and FA metabolism, we next used the gene expression data along with the MCL multi-tissue model to generate flux predictions. Gene expression data was used for generating a context-specific MCL multi-tissue network using an algorithm which uses gene expression data to remove the reactions associated with no gene expression, thereby creating a context-specific metabolic network for the *in silico* T2DM condition simulation. Then, flux variability analysis is performed to find out the reactions without fluxes, which are then removed. The remaining reactions then represent an active context-specific metabolic network. This active model was used to find out the differences between biochemical reaction activity between T2DM and healthy states.

**Figure 7:**
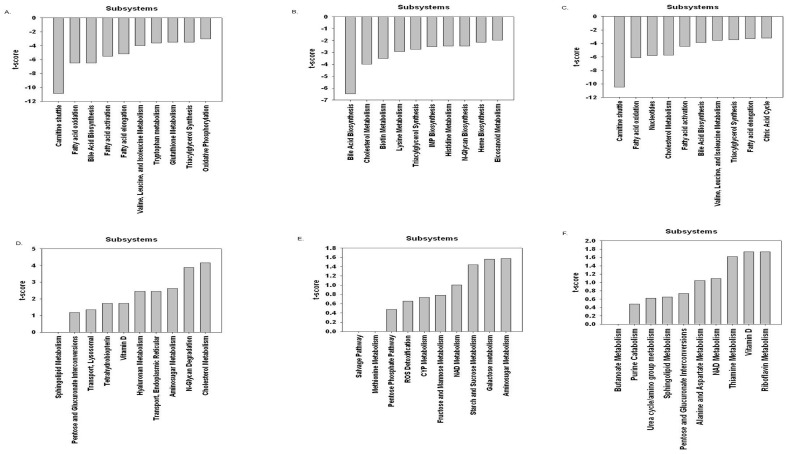
MCL multi-tissue model predictions on different pathways. (A), (B), and (C) represent downregulated pathways (subsystem) for adipose, liver, and skeletal muscle tissues respectively. (D), (E), and (F) represent upregulated pathways for adipose, liver, and skeletal muscle tissues respectively. They are organized by t-score; the more negative the t-score, the more down-regulated the subsystem and the more positive the t-score, the more up-regulated the subsystem.

Figure 7 (A)–(F) represent the data corresponding to each subsystem in the three tissues compared to a hypothetical dataset represented by a random selector. T-scores were obtained for each subsystem to determine the statistical significance of the degree of differential expression. The purpose of organizing the data in this way is to identify particular subsystems affected more greatly by T2DM. This organization scheme helps filter out small subsystems that contain all downregulated reactions but have very few reactions, and therefore do not control the overall behavior of the model.

Cholesterol metabolism is upregulated in adipose tissue (Figure 7 – D) and downregulated in liver tissue (Figure 7 – B) and muscle tissue (Figure 7 – C). This can lead to higher cholesterol level in plasma of T2DM subjects as also observed through actual measurements [54]. The carnitine shuttle is downregulated in adipose tissues (Figure 7 – A) and muscle tissues (Figure 7 – C), and is upregulated in liver tissues (Figure 7 – E). As reported in literature [14], a downregulated carnitine shuttle leads to insulin resistance by triglyceride accumulations in the cytosol of the cells by hampering beta-oxidation. Another interesting observation is the upregulated N-Glycan degradation in adipose tissues (Figure 7 – C) and downregulated N-Glycan biosynthesis in liver tissues (Figure 7 – B). The behavior of these pathways leads to altered N-Glycans structure in the plasma of T2DM subjects [55], [56]. Sphingolipid metabolism is upregulated both in adipose tissues (Figure 7 – D) and muscle tissues (Figure 7 – F), which matches expectations, as ceramide levels in skeletal muscle of T2DM Zucker fatty rat were found normal [57], [58]. However, there is no known relation between T2DM and skeletal muscle ceramide level [59].

It is important to note that in most of the research work done on studying behavior of these subsystems deal with studying differences in gene expression in the two physiological conditions; however, in this study we present differences in fluxes in the biochemical reactions in the two physiological conditions (T2DM vs healthy). Figures S1–S15 in File S1 show percentage of reactions retained by subsystems in the three tissues, fold change analysis in the three tissues, OMIM data analysis including the random selector along with zoomed in version, and robustness analysis results. File S2 provides direction of flux changes (upregulated or downregulated) in different subsystems along with the t-scores in adipose, skeletal muscle, and liver tissues in three individual worksheets corresponding to each tissue type. In the former method of study, if genes associated with few reactions in a subsystem are downregulated, then most of the subsystem will be downregulated because of the steady-state condition. However, in subsystems with a combination of upregulated and downregulated genes, the whole subsystem behaves unpredictably, so the flux results can help decipher such ambiguous situations clearly.

## Discussion

In the present study, microarray technology was used to elucidate the relation of free fatty acid and branched-chain amino acids levels under T2DM and normal condition and the gene expression profiles in three different tissues – liver, muscle, and adipose of diabetic MKR mice. The findings from microarray analysis show that MKR mice have a downregulated fatty acid oxidation profile in muscle and adipose tissues. In liver, the entire fatty acid oxidation profile is not downregulated. On the contrary, fatty acid biosynthesis in liver tissues is considerably upregulated as shown in figure 6. Therefore, a possible explanation for the higher circulating free fatty acid levels in the diabetic MKR mice is the upregulated biosynthesis of fatty acids in liver and considerable lower oxidation of the fatty acids in the muscle and adipose tissues of the animals. Gene expression for branched-chain amino acids metabolism is significantly downregulated too. However, mere downregulation of gene expression does not imply downregulation of the flux through these pathways. Moreover, using gene expression data alone doesn't clearly predict how fast or slow a biochemical reaction proceeds. However, incorporating metabolic flux results, based on such computational models, greatly enhances the understanding of reaction fluxes.

Wang et al. [60] pointed out that comparisons using only enrichment statistics with gene expression data provides far fewer predictions as given by model based approaches. There are a larger number of pathways that can be identified using the model based approach as compared to using gene expression data alone.

Subsequently, an algorithm for building a multi-tissue model is presented in the current paper. The algorithm presented in this paper uses as few linear programming solutions as possible to achieve an optimal solution, so that the solution can be found in a reasonable amount of time. The model provides users with the ability to predict changes in metabolite levels in the medium after deleting specific metabolic genes, as well as the ability to predict changes in metabolite concentrations in the plasma/medium after applying fold changes to specific genes. Other capabilities include using a quadratic programming solver to apply experimentally derived fluxes to specific reactions in the model and determining the remaining fluxes to examine the behavior of the overall model in given by the experiment [17].

The positive aspect of this approach is that metabolic fluxes can be predicted; this is important because metabolic fluxes are difficult to measure in mammalian tissues, and metabolic fluxes provide essential information used for characterizing phenotypes of cellular systems. Flux information can help predict metabolic biomarkers in blood/plasma [22], and can potentially help predict causes for certain metabolic disorders. However, due to their qualitative nature, results from these steady state models should be thought of as supplements to experiments; they are best used to help provide potential targets for biological experiments.

Figures S7–S12 in File S1 delineate the results of the fold change analyses by separating the upregulated and downregulated reactions by subsystems (pathways). As mentioned before, it is easier to find out approximately which subsystems are upregulated or downregulated by microarray analysis, but that does not fully explain the physical effects of the fold changes in these genes, in terms of changes in the metabolic fluxes. The results show a significant downregulation of branched-chain amino acid metabolism and fatty acid oxidation in muscle and adipose tissues (there is some down regulation of branched-chain amino acid metabolism in the liver tissue, but it is less apparent than in the other two tissues). There are more downregulated reactions than upregulated reactions. Changes in gene expression can only be assumed to affect relative enzyme levels, which can only affect upper and lower flux bounds; therefore, we can assume that more downregulated reactions are affected than upregulated reactions because a decrease in the flux bounds represents more restriction than an increase in flux bounds. This is because reactions with increased flux bounds can still be forced to have lower fluxes by adjacent downregulated reactions, whereas reaction with decreased flux bounds cannot be forced to have higher fluxes by adjacent upregulated reactions.

Statistical significance of the differential regulation of the subsystems was calculated by using a two sample t-test (assuming the second sample is a randomly generated set of upregulated, downregulated, and unchanged reaction predictions and is of the same size as the subsystem) to determine the statistical significance of the subsystem's deviation from random behavior. For example, if the subsystem is 100% downregulated with two reactions, then, intuitively, this is not very statistically relevant because a random selector can make this selection 1/9 of the time (assuming the random selector chooses downregulated, upregulated, and unchanged each 1/3 of the time). Thus, sorting by the t-score of each subsystem provides a better understanding of the extent of differential regulation of the subsystems. Figures S1–S12 in File S1 demonstrate the approximate effect of the gene expression during T2DM on certain subsystems. As expected branched chain amino acid metabolism and fatty acid oxidation have very high t-scores in muscle and adipose tissue. The results regarding the branched-chain amino acid metabolism is consistent with the results available in literature [61]. The downregulation of BCAA metabolism in adipose tissue causes an increase in BCAAs in the circulation, which is shown by the amino acid transport fluxes. This increase in circulating BCAAs causes a decrease in BCAA metabolism in muscle tissue as well [53], which is consistent with results in this study. Newgard et al. have reported increased concentration of BCAA in the plasma of T2DM subjects [10]. Although we didn't have access to *in vivo* methods of metabolic flux measurement, we did find evidences supporting our results. For example, it is reported that gluconeogenesis flux in humans is decreased in Type 2 Diabetes [62], which is also demonstrated in our model. In skeletal muscle, free fatty oxidation flux is reduced implying abnormal mitochondrial function [63]–[66] which is also suggested by our results with reduced fatty acid oxidation in skeletal muscle as well as reduced carnitine shuttle which is responsible for fatty acid transport across mitochondria. In order to compare our findings and the above reported findings, we have reported predictions on the BCAA exchange metabolites. Apart from BCAA, other exchange fluxes predictions also resulted from the analysis, but those predictions' in vivo validity with respect to T2DM is not yet confirmed.

The most negative t-score in adipose and muscle tissues is for the carnitine shuttle subsystem. The ‘Nucleotides’ subsystem is also strongly downregulated in both muscle and adipose tissue. Interestingly, liver tissue displays more upregulation than the other tissues, with the carnitine shuttle subsystem showing upregulation. Thus, the carnitine shuttle subsystem appears to be the subsystem most affected in T2DM. The carnitine shuttle is responsible for transport of long chain fatty acids into mitochondria for oxidation. So, a downregulated carnitine shuttle in adipose and muscle tissues implies lower metabolism of the FFAs in those two tissues. Even though the FFA metabolism is higher in liver because of upregulated carnitine shuttle, the biosynthesis pathway of fatty acids is also elevated (Figure 6). This explains the overall high FFA plasma level in T2DM condition. Other subsystems that appear to be affected strongly include most metabolic subsystems of bile acid metabolism, cholesterol metabolism, and branched-chain amino acid metabolism. These subsystems should be studied in more depth in future studies.

In summary, the integration of microarray data and *in silico* predictions via constraint-based modeling has facilitated better understanding of the reason behind high BCAA and FFA levels in plasma of T2DM subjects. Prior systems biology studies have shown the ability of constraint-based models to predict metabolic biomarkers [22]. Similarly, this model provides the complete set of biomarker predictions generated by the genetic fold change study. These metabolites represent potential biomarkers that can facilitate T2DM studies.

Type 2 diabetes is characterized by two major defects: beta-cell dysfunction and insulin resistance in peripheral tissues. The exact alterations in molecular pathways associated with beta-cell dysfunction in insulin-resistant and diabetic states are not clearly understood. Most of the studies involving T2DM and insulin resistance utilize data from insulin-resistant or diabetic animal models, such as Zucker fatty (ZF) and Zucker diabetic fatty rats (ZDF) [27], high-fat-fed mice [28], muscle IGF-1 receptor–lysine–arginine (MKR) mice [29], [30], and lep/lep mice [31]. A common feature of all these animal models is that all models have manifested insulin resistance and often exhibit islet dysfunction as occurs in the early stages of type 2 diabetes in humans. Therefore, it is, in our opinion, valid to use MKR mice and Zucker diabetic fatty rat data for making predictions on humans' T2DM phenotypes.

In a mammalian system, the specific objective of a particular function of a cell, at a given time and under certain conditions, is difficult to identify, whereas in unicellular systems, the objective function can be assumed to be the maximization of cell growth. This is because mammalian systems are much more complex, especially when attempting to define models for different tissues, because each tissue has a different objective. One may consider that there is an over-arching objective function when considering a system that defines the overall human body as a linear combination of partial Recon1's associated with each tissue, but this again is difficult to define, because the objective of maximizing biomass in unicellular organisms only applies during the exponential growth phase. Thus, maximizing biomass in mammalian systems may be applicable, but only during the growth phase of the mammal.

## Materials and Methods

### B.1 Animal Studies

All animal study protocols were approved by the Mount Sinai School of Medicine Institutional Animal Care and Use Committee (IACUC). Mice were housed in The Mount Sinai School of Medicine Center for Comparative Medicine and Surgery, an Association for Assessment and Accreditation of Laboratory Animal Care (AAALAC) and Office of Laboratory Animal Welfare (OLAW) accredited facility, where animal care and maintenance were provided.

Male 10-weeks old FVB/N – MKR mice were used for the microarray studies. Generation and characterization of MKR mice have been described elsewhere [32]. Mice were kept on a 12-h light/dark cycle, they were allowed free access to diet (Picolab rodent diet #5053) and fresh water. Death of the mice was caused by subjecting them to CO_2_. Liver, Skeletal muscle, and fat tissues were flash frozen in liquid nitrogen, stored at −80°C, and shipped on dry ice to NIDDK/NIH facility for further processing.

### B.2 RNA Sampling

Total RNA from the liver, fat, and skeletal muscle tissues was isolated for three biological replicates for both diseased and healthy animal subjects. Qiagen's Microarray Tissue Mini Kit (Qiagen GmbH, Germany) was used for RNA isolation purpose according to the manufacturer's instructions. Purified total RNA was quantified using a spectrophotometer (Thermo Scientific Ltd). The absorbance values at 260 and 280 nm were used for assessing the quality of the sample. Only the samples with greater than 1.80 260/280 absorbance ratio were used for microarray analysis [67].

### B.3 Microarrays procedure, Statistical analysis and Biological Inference

RNA quality was tested using bioanalyzer (RNA Nano assay in the Expert 2100 software, Agilent Technologies, CA) and RIN (RNA Integrity Number) values were above 8.0 for all the samples. 100 ng of RNA from each sample was amplified to generate cDNA using NUGEN Applause WT – Amp ST system (NuGEN Technologies, CA), according to the manufacturer's instructions. 2.5 µg of cDNA was fragmented and biotinylated using Encore Biotin module (NUGEN Technologies, CA). Resultant sample mix with hybridization reagents (Affymetrix Inc. CA) and injected into Affymetrix Mouse Gene 1.0 ST arrays and incubated for 18+2 hours in hybridization oven rotating at 60 rpm at 45°C (Affymetrix Inc., CA). Arrays were processed using Affymetrix 450 Fluidic stations using wash and stain kit (Affymetrix Inc.). Chips were scanned using Affymetrix GeneChip scanner 3000 operated by Gene Chip Operating Software, version 1. 4 (GCOS 1.4) and generated.CEL.CHP and RPT files. To access the efficiency of cDNA synthesis Poly A controls (dap, lys, phe, thr- Affymetrix Inc.) was spiked to the samples and hybridization controls (bioB, bioD, bioC and Cre, Affymetrix Inc.) were added to monitor labeling efficiency according to the manufacturer's instructions.

The microarray raw data was analyzed using software Partek genomics suite, version 6.3 Copyright 2008 Partek Inc., St. Louis, MO, USA. Raw data were subjected to Robust Multichip Average (RMA) quantile normalization to remove biases introduced by technical and experimental effects. All expression data were log base 2 -transformed to get near normal distribution for accurate statistical inference. Quality control by visualizing the data using Principal Component Analysis cluster plot ensured that no outliers were included for the analysis. Next, two-way ANOVA analysis was performed to obtain a set of differentially expressed genes. A filter of P-value <0.05 and Fold-change >1.5 times was applied to get the significantly differentially expressed genes list. The results of the microarray analysis have been deposited to National Center for Biotechnology Information (NCBI) repository and can be accessed with Gene Omnibus Expression accession: GSE51866.

The significantly differentially expressed genes list was exported to – Ingenuity Pathway Analysis (Ingenuity Systems, www.ingenuity.com) for finding biological inference. Ingenuity Pathway Analysis is an online software used to study relationship between genes, proteins, and biological reactions. More technical details about the Ingenuity Pathway Analysis' capabilities can be accessed from the Ingenuity Systems website. Statistically significant genes from Partek analysis were overlaid on the Ingenuity Pathway Analysis global molecular network, which is based on information from other databases such as KEGG, HumanCyc, etc. in the Ingenuity knowledge base after applying a filter on species type (mouse) and tissue type (e.g. adipose).

### B.4 Tissue-specific Model Building Algorithm

Published, detailed reconstruction of human metabolism (Recon1) was downloaded from the BiGG database in SBML format [68], [69]. The models were created, maintained, and altered using the COBRA toolbox in MATLAB version 2010a [70]. A three tissue version of Recon1 was generated by adding prefixes -‘A:’, ‘H:’, and ‘M:’ to each reaction name and suffixes - [Adp], [Hep], and [Msc] to each metabolite for adipocytes, hepatocytes, and skeletal muscle tissue respectively. The extracellular compartment is shared between all three tissues and only one set of reactions comprising of only extracellular metabolites was maintained in the MCL multi-tissue model. After removing all reactions associated with dead-end metabolites from the model, the size of the three tissue version of Recon1 becomes 6644 reactions with 4103 metabolites.

The algorithm for trimming down the general three tissue version of Recon1 is built by minimizing the number of linear programming problems needed to trim the reconstruction [17], [22]. The algorithm contains three sets of reactions: a high-confidence set, a medium-confidence set, and a low-confidence set. The high confidence set of reactions was created based on literature results containing confirmed protein expression in the three tissues and were mapped to specific reactions in Recon1 for specific tissue types [71]–[101]. The medium confidence set of reactions was obtained from tissue-specific data from publically available databases HPRD [102]–[104], UniProt [105], and Brenda [106] and their online databases with tissue-specific data [107]–[109]. The low confidence set of reactions was the list of remaining reactions that were not in either the high or medium confidence sets.

The overall goal of this algorithm is: (1) maintain all high confidence reactions and (2) maximize the number of medium confidence reactions minus 0.5 multiplied by the number of low confidence reactions in the final trimmed version of the reconstruction. Statement (2) can be considered the objective function of the algorithm as shown below –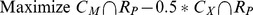



The value 0.5 reflects equal probability of obtaining the most parsimonious model as well as including a maximal number of moderate probability reactions in the partial model using above algorithm [17].

R_p_ is a partial subset of reactions from Recon1 that defines the solution space for the tissue-specific reconstruction, C_M_ and C_X_ are the medium and low confidence set of reactions, respectively. The algorithm makes more efficient use of all of the information gained from a single linear programming (LP) solution. Every reconstruction can be broken down into elementary flux modes; these can be thought of as the simplest possible flux distributions. Therefore, every reconstruction is a linear combination of elementary flux modes, and thus, each linear programming solution is a smaller linear combination of elementary flux modes. Ideally, the algorithm would identify all elementary flux modes in a reconstruction or even LP solution and find the optimal combination from the elementary flux modes, but as of now, this is computationally infeasible due to combinatorial explosion [110]. Elementary flux modes have been determined for smaller networks, but cannot be found for more complex networks like Recon1. However, LP solutions represent feasible flux distributions that are typically much smaller than the size of the final networks. Final networks can also be thought of as linear combinations of all of these possible linear programming solutions. Therefore, the algorithm pre-loads a very large matrix with information on around 10,000–20,000 randomly generated flux distributions represented by linear programming solutions. The randomization of the solutions is the randomization of the objective functions of the associated linear programming problems. The algorithm randomly selects a few high confidence and medium confidence reactions to be maximized in the objective function; low confidence reactions will never be in the objective function. Then, a Boolean vector is created which describes the activity of each reaction in the network (1 for active and 0 for inactive). This vector represents one flux distribution and also represents one column in the large matrix of random flux distributions (the large matrix is named fdMatrix). The rows of each Boolean column vector, and consequently the matrix, represent distinct reactions in the general network. The creation of this matrix represents the second step of the algorithm.

The next step of the algorithm consists of providing scores for each flux distribution. The scores are calculated by subtracting half the number of active low confidence reactions from the number of active medium confidence reactions. If a score is positive, it means that adding the reactions associated with this flux distribution will add to the value of the objective function of the algorithm. Scores are calculated for each column in the matrix. This marks the beginning of the third step of the algorithm. This step involves addition of flux distributions associated with the high confidence set of reactions. It starts by ordering the list of scores associated with the flux distributions from high to low values. Then, the flux distribution with the highest score is checked for any active high confidence reactions; if there are no high confidence reactions, then the flux distribution with the second highest score is checked for high confidence reactions. This procedure continues until the highest scoring flux distribution containing at least one high confidence reaction is found. All of the active reactions within this flux distribution are added to a final list of reactions. Also, any high confidence reactions added to the final list of reactions are removed from the list of remaining high confidence reactions. Then, the rows of the fdMatrix which are associated with the set of active reactions that were recently added to the final list of reactions are set to rows filled with zeros. This is done because these reactions are now in the final list of reactions and therefore do not contribute to the objective score anymore. Changing the values in the rows changes the scores associated with each column/flux distribution in fdMatrix, thus the scores must be re-calculated to reflect the changes in the rows. These re-calculated scores are sorted again to reveal the columns/flux distributions with the highest scores. Then, the highest scoring column/flux distribution with at least one reaction that is in the list of remaining high confidence reactions is selected, and the whole cycle repeats itself. This cycle is repeated until there are no reactions left in the list of remaining high confidence reactions, which means that all of the high confidence reactions were added to the list of final reactions, which is one of the requirements of the algorithm. Also, it's important to note that the high confidence reactions were added in a way that maximizes the score/objective function of the algorithm.

The next step of the algorithm involves adding other flux distributions that increase the value of the objective function. After adding all of the high confidence reactions, the number of medium confidence reactions added to the reconstruction was optimized. The process for adding flux distributions that adds a positive value to the objective function is very similar to the process that added all of the high confidence reactions. Each of the remaining flux distributions is assigned an objective score based on the number of medium confidence reactions and low confidence reactions remaining within that distribution. These scores are sorted to find the distribution with the highest objective value. If the highest objective score is above zero, then the reactions of that distribution are added to the reconstruction. Then, the rows that correspond to the reactions that were just added are filled with zeros to prevent double counting toward the objective function. This process is repeated as long as the highest objective score is greater than zero. This means that reactions are added only if the corresponding distributions increase the objective function, and reactions are no longer added when the objective function cannot be increased by the randomly created distributions, meaning that the objective function is at a maximum for this particular set of flux distributions.

Then, this whole process is repeated again; a new set of random flux distributions is created based on the partial model this time (the distributions were created from the general model the first time), and reactions from the high confidence list are added along with their associated reactions determined by the flux distributions, and medium confidence reactions are optimized. As part of the model building algorithm during the reconstruction process, maximizing the flux to ascertain activity/inactivity of a reaction is needed. A random flux distribution is used only in the reconstruction process which is subjected to our algorithm to find out whether each reaction adds to biological meaning or not. This process repeats itself until the model doesn't change in size from one iteration to the next. The time required to solve a problem associated with a three tissue system varies from 0.05–0.1 seconds. The number of reactions maximized in each iteration is variable; in the first iteration, all reactions are maximized. In the second iteration, all reactions that had zero flux in the solution to the first linear programming iteration are maximized, and so on. This continues until either all reactions are proven to carry a flux (have a non-zero solution to any linear programming problem), or some set of reactions are proven to be unable to carry flux (the set of reactions is maximized/minimized and no flux profiles result). The latter is explicitly proven by performing distinct linear programming problems and maximizing/minimizing each reaction in the set individually as explained in f tissue-specific model building algorithm.

### B.5 Validation Procedure

Robustness analysis of the algorithm was performed by generating 5 replicate models with starting point as 5 different randomized stoichiometric matrices. All 5 of the replicate models were similar in size (Figure S15 in File S1), thereby validating the robustness of the algorithm. Further validations of the model are described in the following subsections.

#### B.5.1 OMIM gene deletion analysis

The first validation procedure uses similar approach as used in studies by Shlomi *et al.* The validation tests the effect of deleting genes *in silico* versus experimental data. The experimental phenotypic effect of deleting genes is available in the public database – OMIM [111]. The known biomarkers of the amino acid-associated disorders compiled above were manually extracted from the disease description field in the OMIM database. This set of disorders was further filtered to include only the disorders that were reported to show a concentration change in at least one of the model's boundary metabolites. This resulted in a final set of 17 disorders that composed the validation set. In this validation, disorders that are known to increase or decrease amino acid levels in the blood were considered. This is because the calculated steady state models can only allow accumulation in the extracellular (blood) compartment.

After mapping OMIM disorder identifier to specific human genes, reactions associated with the affected genes were found. Then, those reactions were artificially “turned on” by equating the lower bounds of the associated reactions to 1. This means that a flux with zero value through the affected reactions is not permitted. The purpose of forcing the reactions to be active is to model a situation in which these reactions are used; this will provide a greater contrast for when the reactions are removed (if zero flux is still allowed, then the model may not use the affected reactions and therefore the comparison would be between reactions that may not be used and reactions that are deactivated). After the lower bounds are changed, flux variability analysis is performed on the model [112] providing a minimum and maximum flux allowed by the solution space of the model. Then, the two sets of flux bounds are compared to determine if the reactions are upregulated, downregulated or unchanged. The reactions are considered either upregulated or downregulated if the change in flux bounds is greater than a threshold, or less than the negative of that same threshold. The change between the sets of flux bounds is calculated by:

For reaction ‘i’:

Disease reaction flux < wild-type reaction flux if,
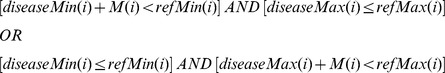



Disease reaction flux > wild-type reaction flux if,
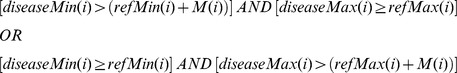



Where:
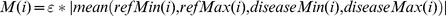



The set of reactions represented by the variable ‘*i*’ are the set of exchange reactions of metabolites that have been identified in the phenotype descriptions of the OMIM database. In this validation, they are amino acids. The variables diseaseMax and diseaseMin are the flux bounds of the amino acids in the disease version of the model with particular genes knocked out. The variables refMin and refMax are the flux bounds of the amino acids in the healthy model that has no genes knocked out. The variable ε represents the threshold value used for the receiver-operator characteristic (ROC) curves.

The results of this validation are depicted via ROC curves in the Results section. In this simulation, the threshold value is varied to distinguish between very large changes in flux bounds and very small changes in flux bounds. Thus, for a given threshold value and disease, upregulated and downregulated predictions are made for each amino acid exchange reaction. These predictions are checked against the known phenotype of that specific disease. This generates a list of true positive, false positive, true negative, and false negative predictions for the specific disease at a particular threshold. This analysis is repeated using the same threshold value for each disease in the validation. All of the predictions from each disease are added together, to provide a number of true positives (TP), false positives (FP), true negatives (TN), and false negatives (FN) for that particular threshold value. Then, the false positive rate (FPR) and the true positive rate (TPR) are calculated as below.
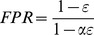


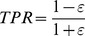



Where, 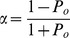; P_o_ is proportion of unchanged metabolites in the data; 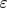 is the threshold, defined above.

TPR  =  true positive rate; (number of true positives)/(number of true positives + number of false negatives).

FPR  =  false positive rate; (number of false positives)/(number of false positives + number of true negatives).

These values for FPR and TPR are calculated for a range of threshold values; typically from 0 to 1000, and plotted with FPR on the x-axis and TPR is on the y-axis. The area under the curve (AUC) of this plot is the indicator of the quality of the reconstruction network. An AUC of 0.5 represents a random classifier; any AUC over 0.5 represents a system that is better than a random guessing.

#### B.5.2 T2DM gene expression fold change analysis

The gene expression of adipose, liver, and muscle tissue for MKR mice model was examined. The MKR mouse model was developed by over-expressing the IGF-I receptor in skeletal muscle [113]. Statistical tests were used to identify genes that were significantly upregulated or downregulated (p-value <0.05). These genes were mapped to Entrez gene IDs, and subsequently mapped to the steady-state model. This identified specific reactions that were either upregulated or downregulated due to differential gene transcription in type II diabetes. For reactions that had both upregulated and downregulated genes mapped to them, regulation status of the reaction was determined by summing the number of up and downregulated genes that map to that specific reaction in question and compare the results. This is only done when multiple gene IDs from the microarray data map to the same Entrez gene ID; this is not performed in the case where multiple distinct gene IDs map to the same reaction through gene-to-reaction maps. In the latter case, the gene-to-reaction logical mapping is used to determine the regulation status.

Transcription levels of genes do not directly correspond to reaction fluxes, but a few assumptions can be made to demonstrate the effect of the levels. The main assumption is that relative gene transcription levels correlate with relative protein concentrations. With this assumption, the effect of increased or decreased protein concentration manifests itself in the upper and lower bounds of the reactions that are associated with the protein. The reason for this is because enzyme concentration only affects V_max_ in enzyme kinetics equations. V_max_ is equal to k_cat_ multiplied by enzyme concentration; therefore, a lower enzyme concentration decreases the maximum reaction velocity (and minimum reaction velocity, if the reaction is reversible). Reactions that are determined to be affected by gene regulation have upper bounds initially set to 100 and lower bounds initially set to −100 or 0 depending on reversibility. Unaffected reactions have normal reaction bounds (upper bounds are 1000 and lower bounds are −1000 or 0). Initial FVA is then performed. Then, upregulated reactions have bounds doubled downregulated reactions have bounds halved. Then, FVA is performed, and resulting bounds are compared to initial FVA results for comparison and analysis. The reasoning for initially changing affected reaction bounds from 1000 to 100 was to magnify the effect of upgregulating reactions. If reaction bounds of affected reactions are kept at normal 1000 values, then doubling the reaction bounds due to upregulation may not display downstream upregulation or display the far-reaching effects of gene upregulation. The lower bound and upper bound for exchange reactions are 1 and 1000 respectively.

The first step is to identify all upregulated and downregulated reactions, and change the associated bounds to one-tenth of the initial value. This is done so that the effect of increases in reaction bounds due to upregulation will have an effect in a steady-state model. Then, FVA is performed on the model to establish a control set of bounds. After that, the bounds of upregulated reactions are doubled and downregulated reactions are halved. Then, FVA is performed again to generate a disease-state set of bounds. Then, the difference between the two sets of bounds is determined by the following:

For a given reaction ‘
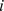
’;
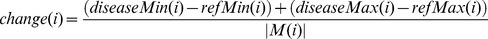




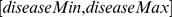
 represent the lower and upper bounds of the disease set.


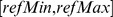
 represent the lower and upper bounds of the control set.


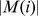
 is the absolute value of the mean of all four bounds for reaction ‘i’.

Literature results were obtained for specific concentrations of amino acids in the plasma for T2DM rat versus healthy rat. The effect on each of the transport fluxes of each amino acid were considered to determine an increase or decrease in concentration in the plasma. This was done instead of simply inspecting the exchange reactions for each amino acid because the full effect of the gene expression changes may not be observed on an exchange reaction if an unaffected pathway exists that involves the exchange reaction in question. Taking all transport reactions into account generates a more complete picture of the uptake or secretion of metabolites.

## Supporting Information

File S1 **Supporting figures. Figure S1. Adipose Chart 1**: Percentage of Reactions Retained by Subsystem in Adipose Tissue – 1. **Figure S2. Adipose Chart 2**: Percentage of Reactions Retained by Subsystem in Adipose Tissue – 2. **Figure S3. Liver Chart 1**: Percentage of Reactions Retained by Subsystem in Liver Tissue – 1. **Figure S4. Liver Chart 2**: Percentage of Reactions Retained by Subsystem in Liver Tissue – 2. **Figure S5. Skeletal Muscle Chart 1**: Percentage of Reactions Retained by Subsystem in Skeletal Muscle Tissue – 1. **Figure S6. Skeletal Muscle Chart 2**: Percentage of Reactions Retained by Subsystem in Skeletal Muscle Tissue – 2. **Figure S7. Adipose Fold Change 1**: Fold Change Analysis in Adipose Tissue – 1. **Figure S8. Adipose Fold Change 2**: Fold Change Analysis in Adipose Tissue – 2. **Figure S9. Liver Fold Change 1**: Fold Change Analysis in Liver Tissue – 1. **Figure S10. Liver Fold Change 2**: Fold Change Analysis in Liver Tissue – 2. **Figure S11. Skeletal Muscle Fold Change 1**: Fold Change Analysis in Skeletal Muscle Tissue – 1. **Figure S12. Skeletal Muscle Fold Change 2**: Skeletal Fold Change Analysis in Muscle Tissue – 2. **Figure S13. OMIM gene data analysis**: OMIM data analysis including random selector. **Figure S14. OMIM gene data analysis – closeup**: OMIM data analysis including random selector zoomed in version. **Figure S15. Robustness analysis results**: Table in the figure shows number of reactions in each replicate models and the bar plot gives clear visualization of the same.
(PPT)

File S2 (XLS)
