# Supplementary material for: Multi-Tissue Computational Modeling Analyzes Pathophysiology of Type 2 Diabetes in MKR Mice
Source: PLoS One. 2014 Jul 16;9(7):e102319. doi: 10.1371/journal.pone.0102319 (PMC4100879; doi:10.1371/journal.pone.0102319)
Supplement: File S1 — Supporting figures. Figure S1. Adipose Chart 1: Percentage of Reactions Retained by Subsystem in Adipose Tissue – 1. Figure S2. Adipose Chart 2: Percentage of Reactions Retained by Subsystem in Adipose Tissue – 2. Figure S3. Liver Chart 1: Percentage of Reactions Retained by Subsystem in Liver Tissue – 1. Figure S4. Liver Chart 2: Percentage of Reactions Retained by Subsystem in Liver Tissue – 2. Figure S5. Skeletal Muscle Chart 1: Percentage of Reactions Retained by Subsystem in Skeletal Muscle Tissue – 1. Figure S6. Skeletal Muscle Chart 2: Percentage of Reactions Retained by Subsystem in Skeletal Muscle Tissue – 2. Figure S7. Adipose Fold Change 1: Fold Change Analysis in Adipose Tissue – 1. Figure S8. Adipose Fold Change 2: Fold Change Analysis in Adipose Tissue – 2. Figure S9. Liver Fold Change 1: Fold Change Analysis in Liver Tissue – 1. Figure S10. Liver Fold Change 2: Fold Change Analysis in Liver Tissue – 2. Figure S11. Skeletal Muscle Fold Change 1: Fold Change Analysis in Skeletal Muscle Tissue – 1. Figure S12. Skeletal Muscle Fold Change 2: Skeletal Fold Change Analysis in Muscle Tissue – 2. Figure S13. OMIM gene data analysis: OMIM data analysis including random selector. Figure S14. OMIM gene data analysis – closeup: OMIM data analysis including random selector zoomed in version. Figure S15. Robustness analysis results: Table in the figure shows number of reactions in each replicate models and the bar plot gives clear visualization of the same. (PPT) [file pone.0102319.s001.ppt]

## Slide 1
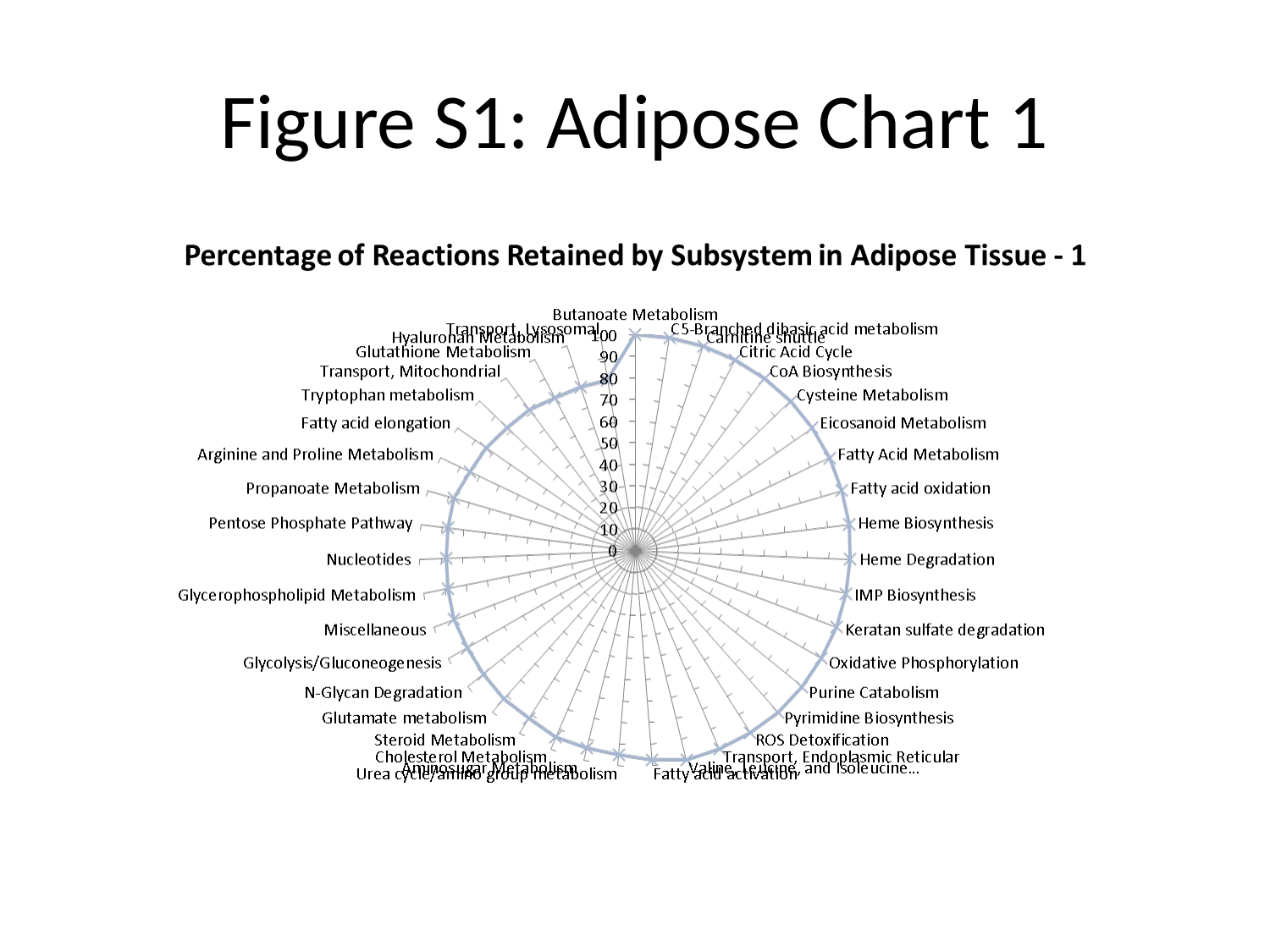

# Figure S1: Adipose Chart 1

## Slide 2
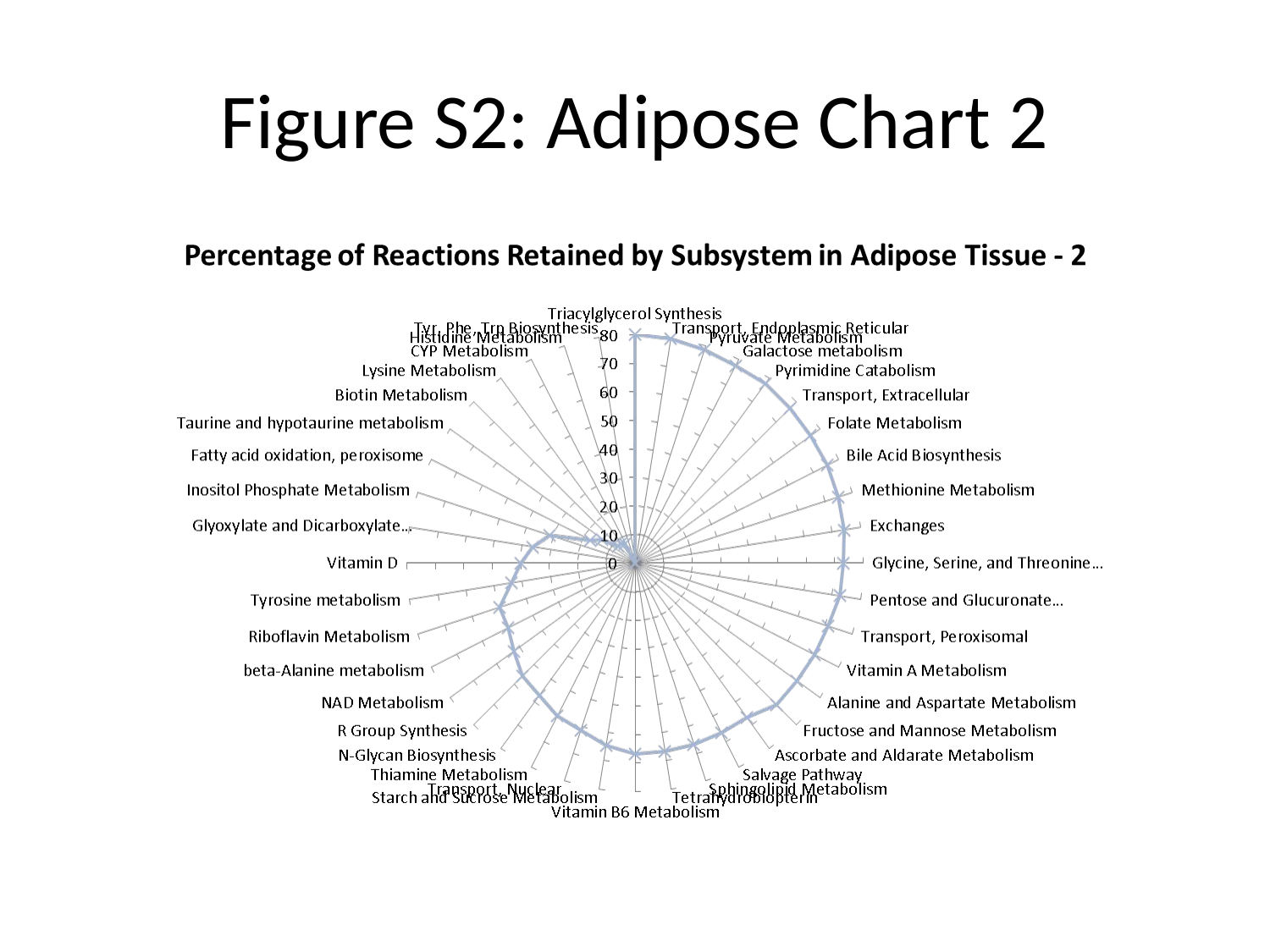

# Figure S2: Adipose Chart 2

## Slide 3
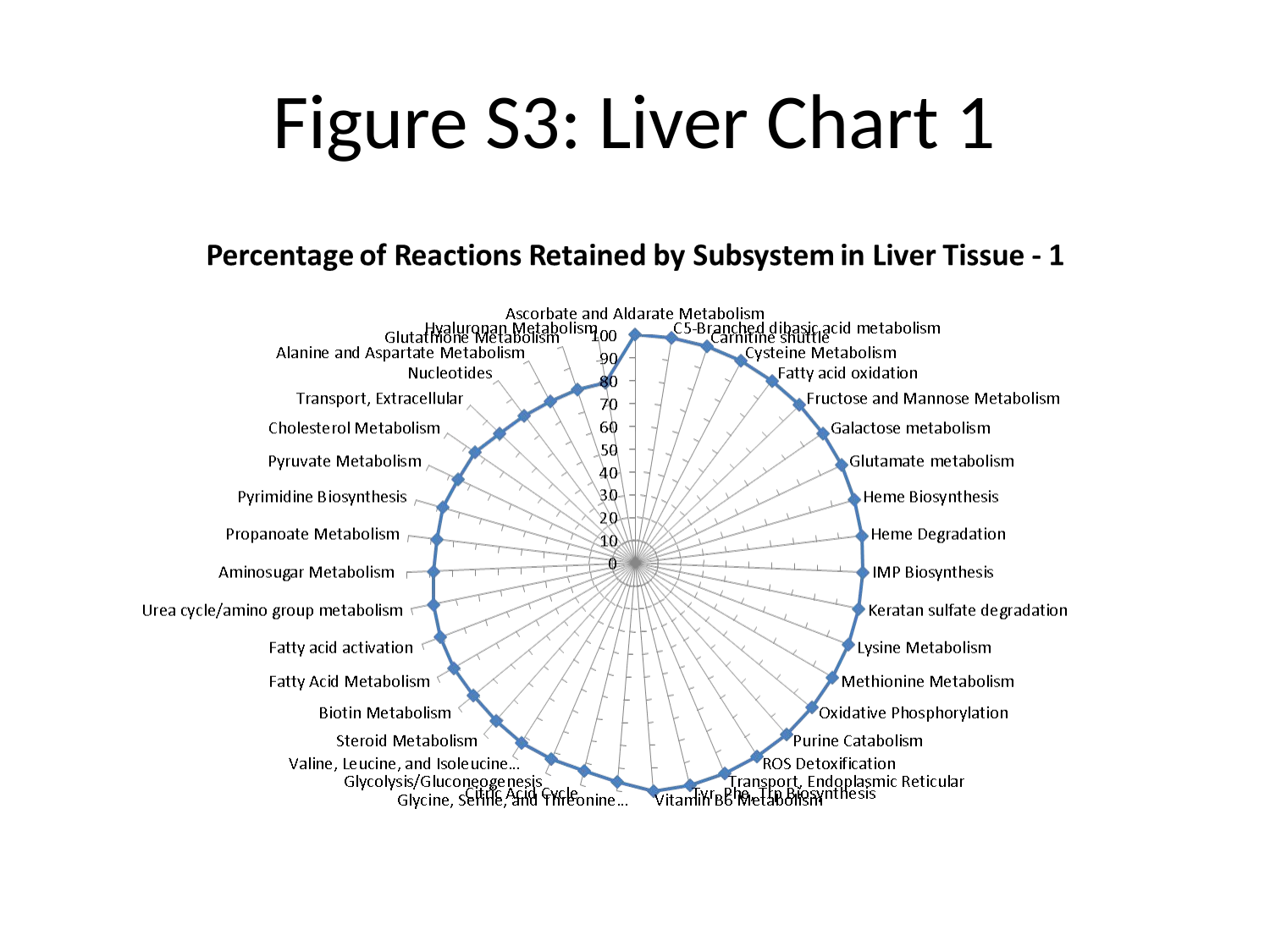

# Figure S3: Liver Chart 1

## Slide 4
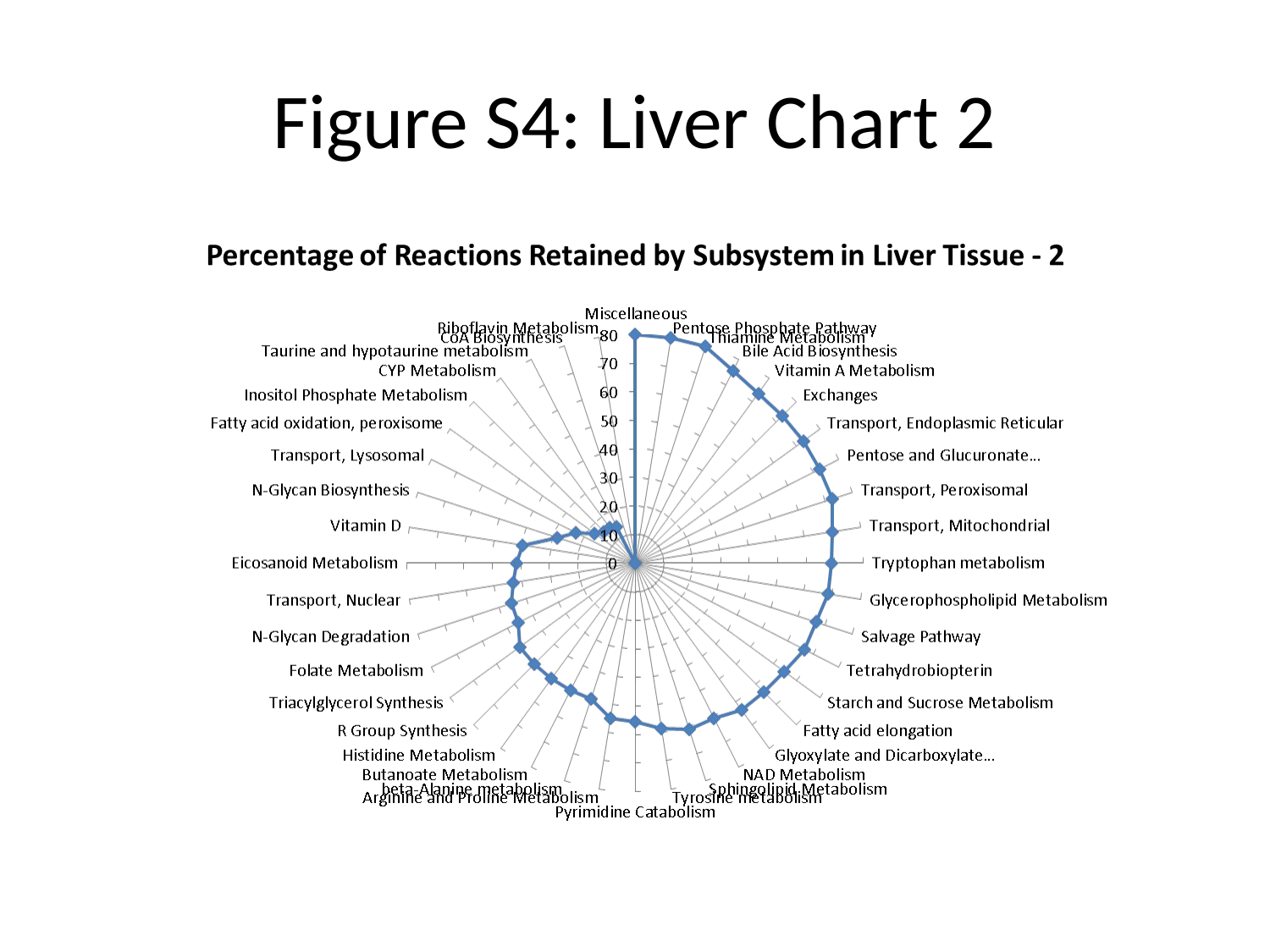

# Figure S4: Liver Chart 2

## Slide 5
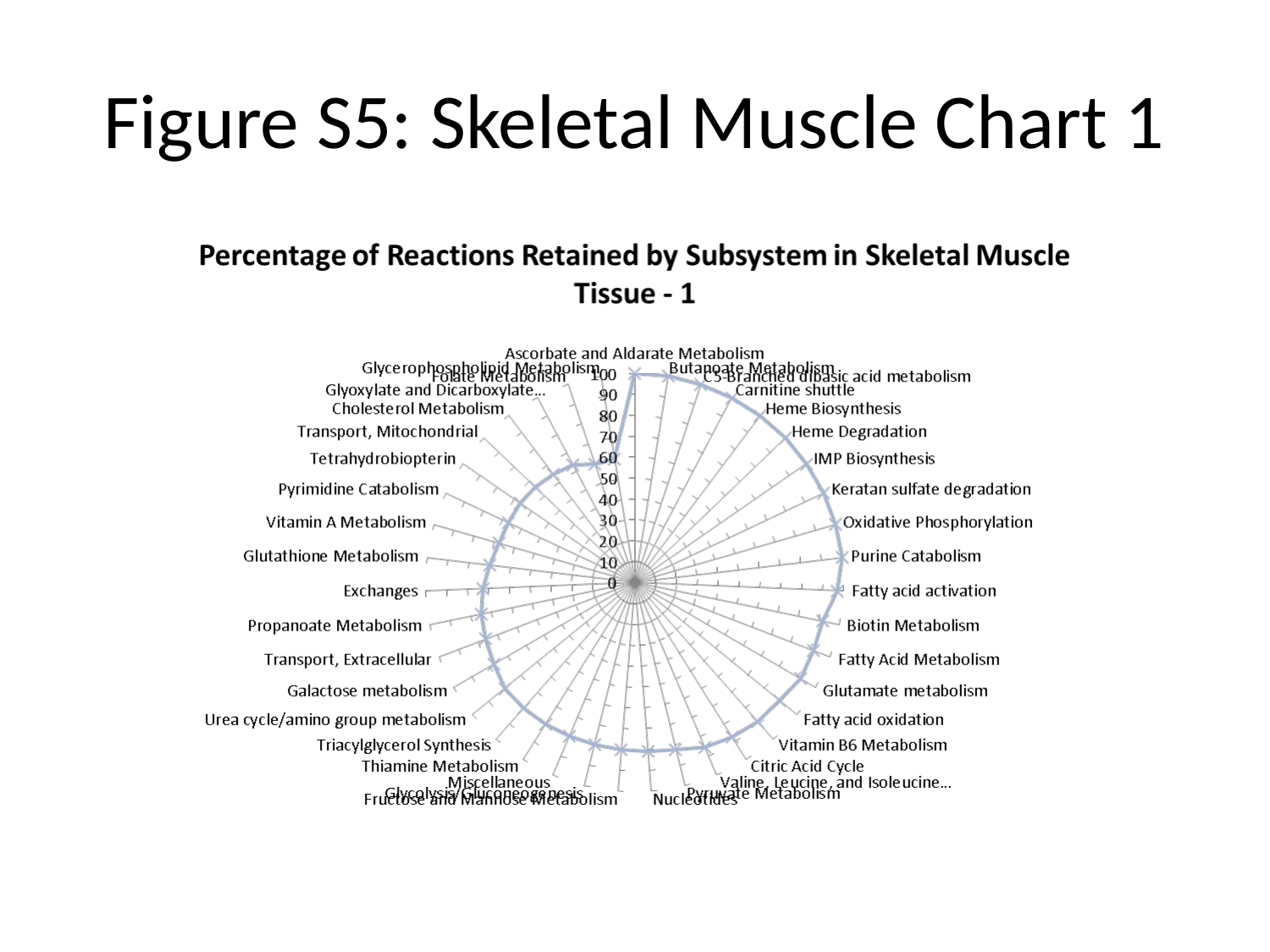

# Figure S5: Skeletal Muscle Chart 1

## Slide 6
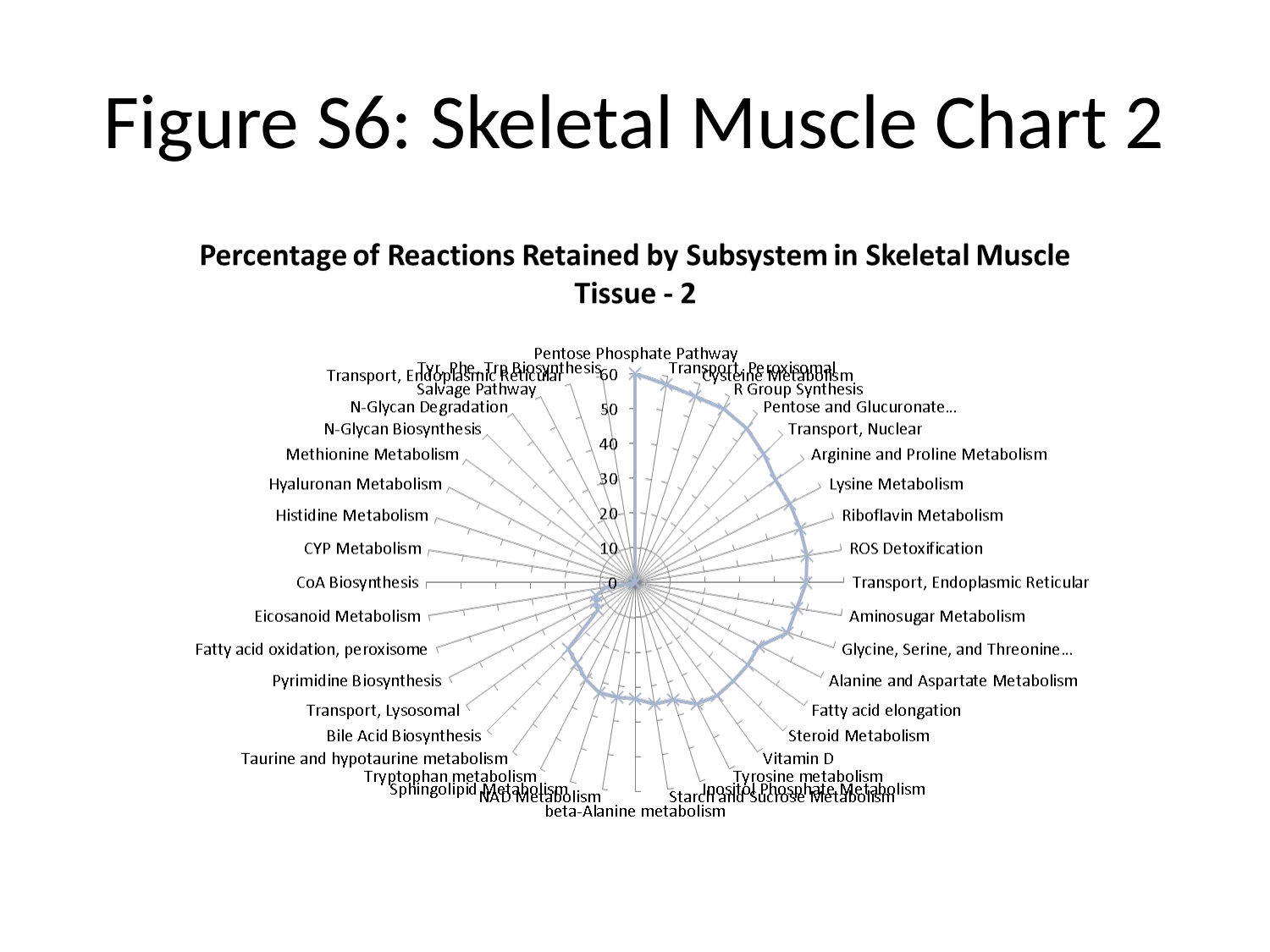

# Figure S6: Skeletal Muscle Chart 2

## Slide 7
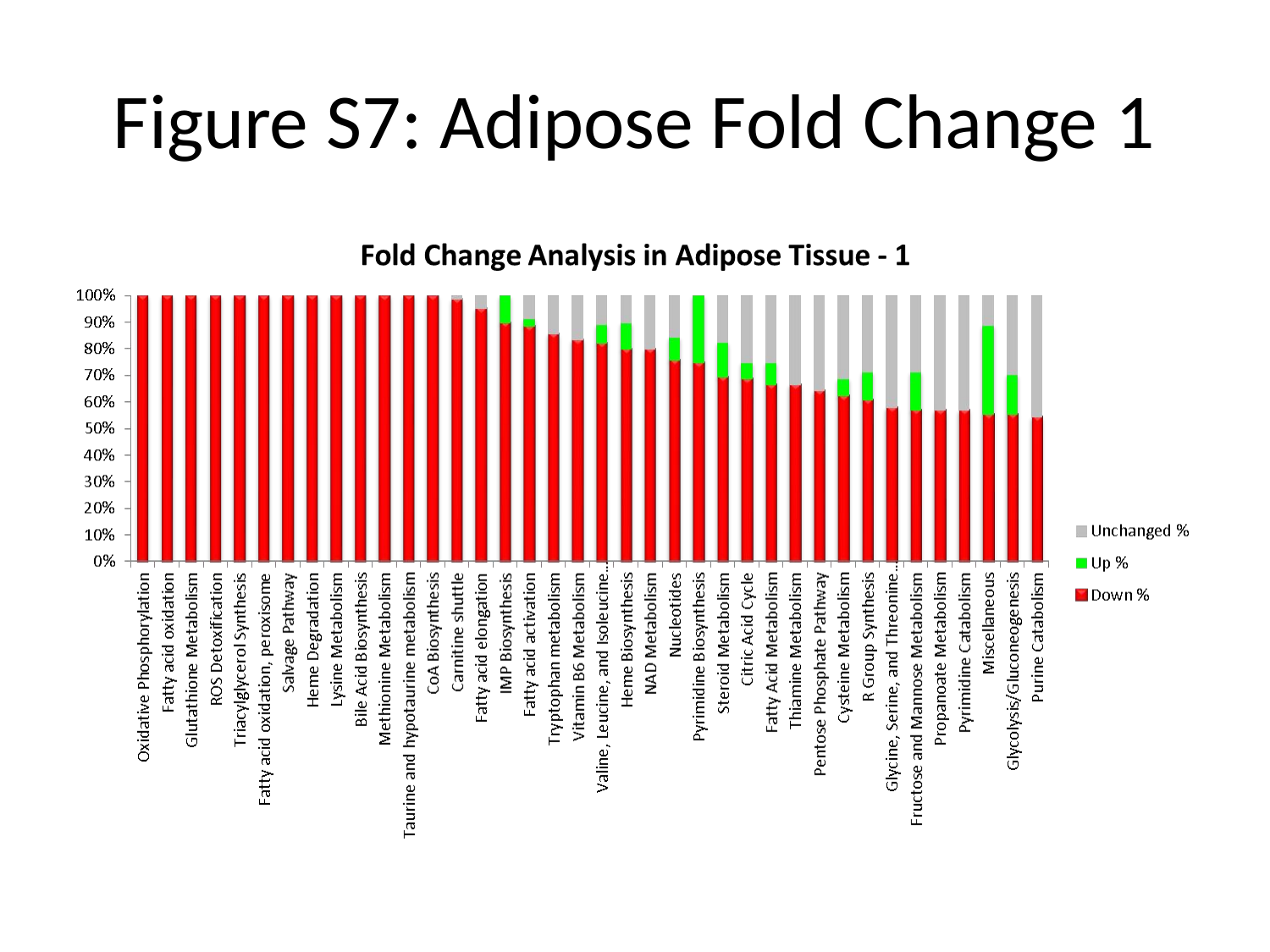

# Figure S7: Adipose Fold Change 1

## Slide 8
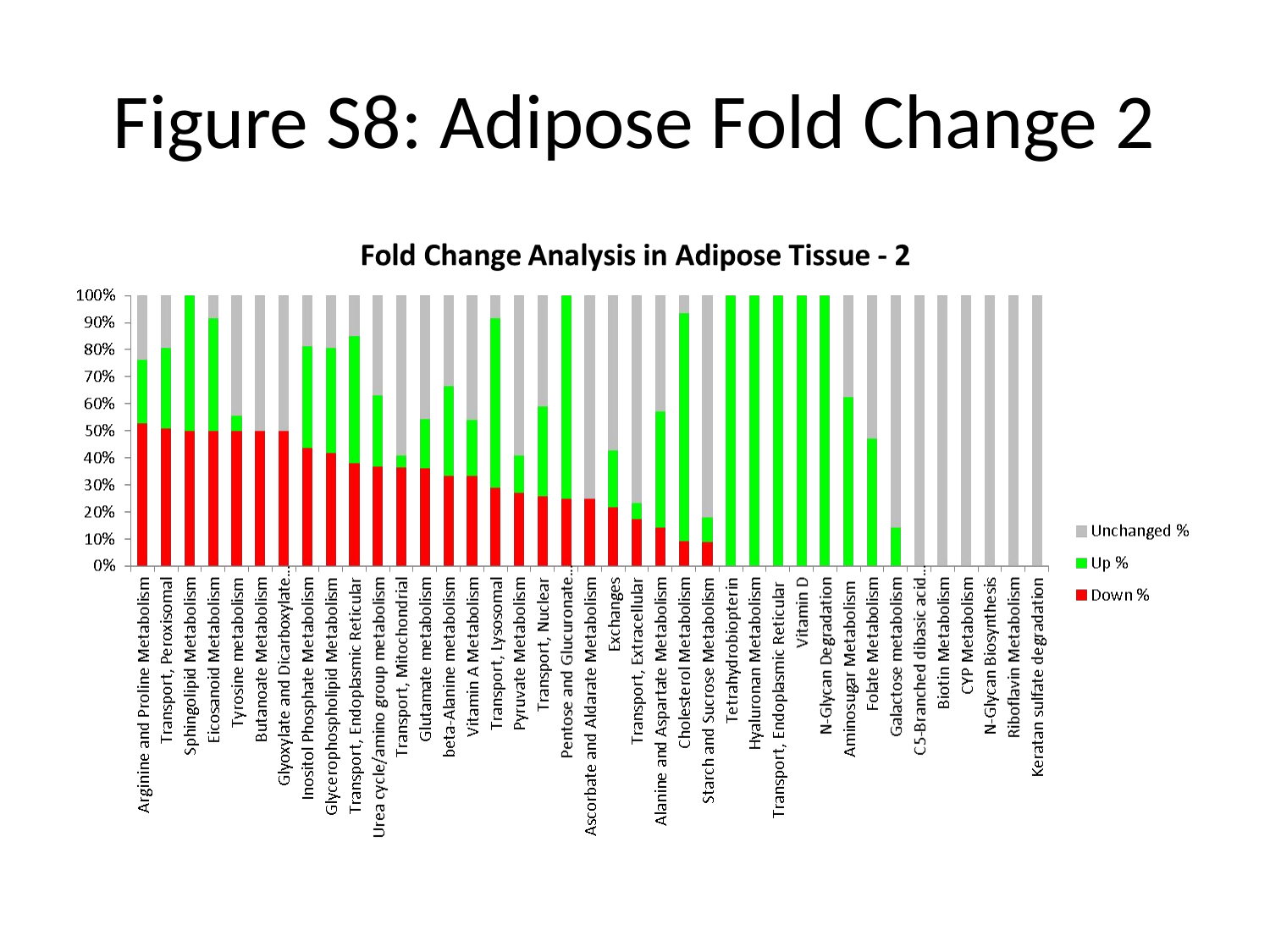

# Figure S8: Adipose Fold Change 2

## Slide 9
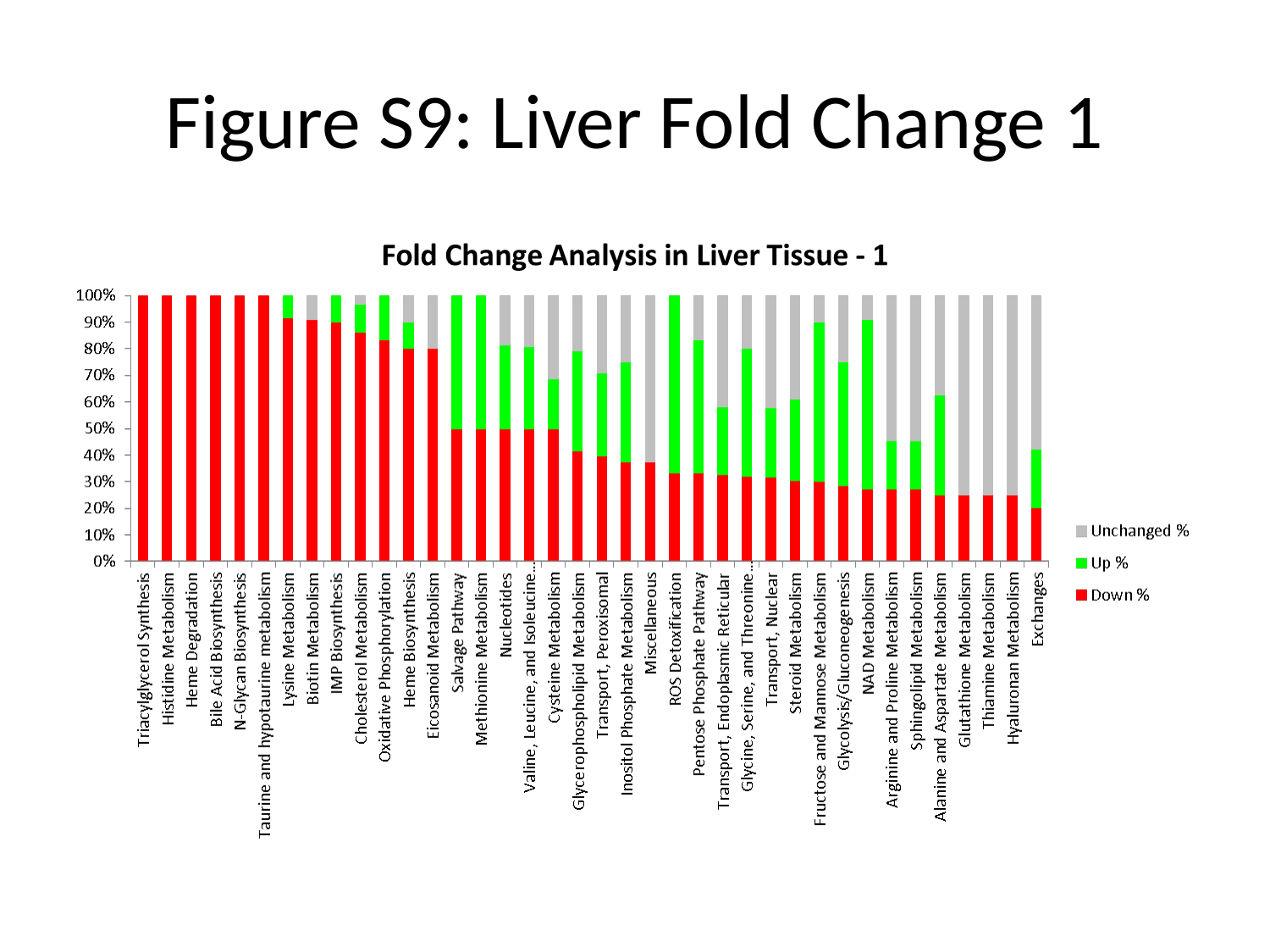

# Figure S9: Liver Fold Change 1

## Slide 10
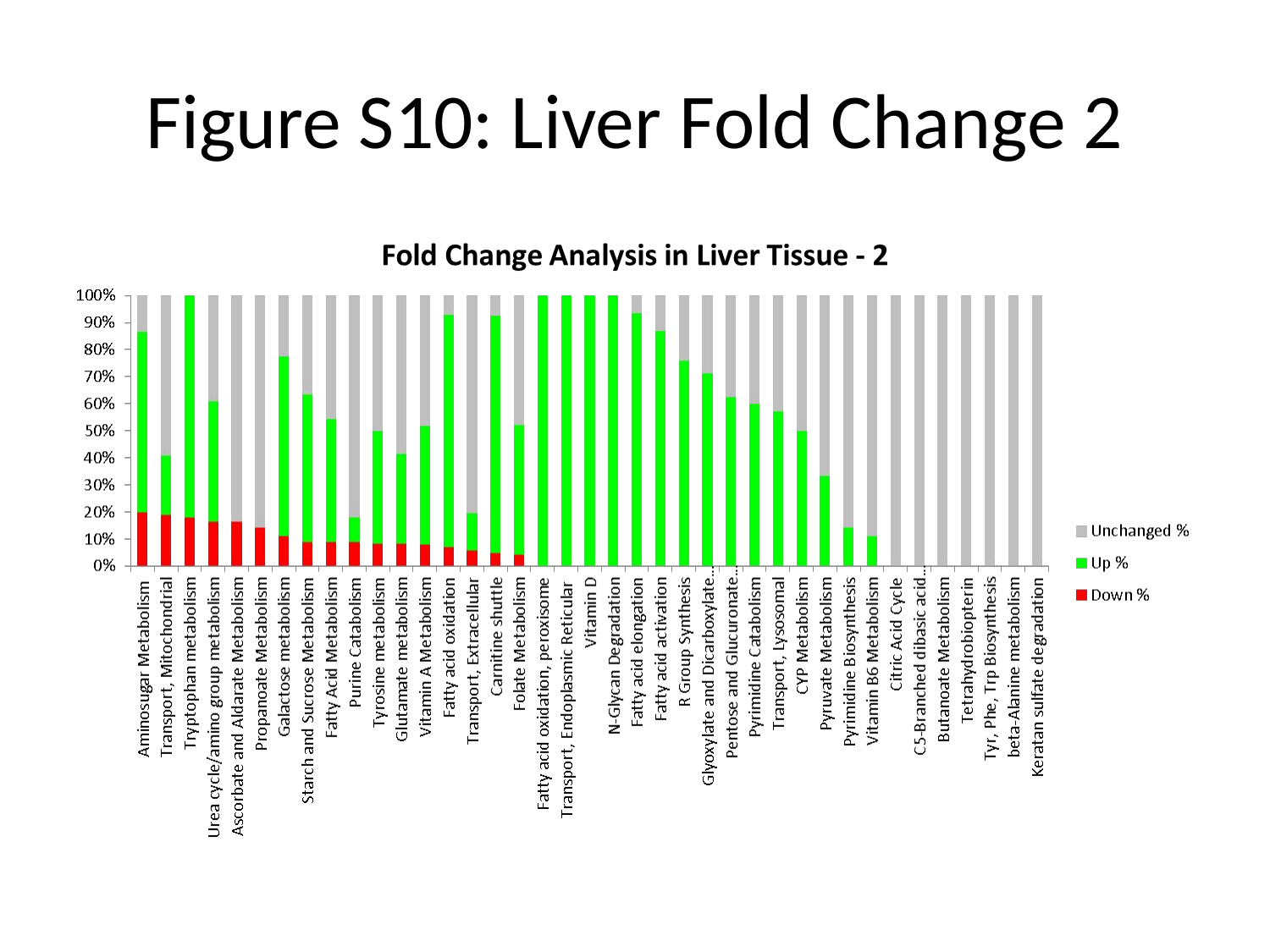

# Figure S10: Liver Fold Change 2

## Slide 11
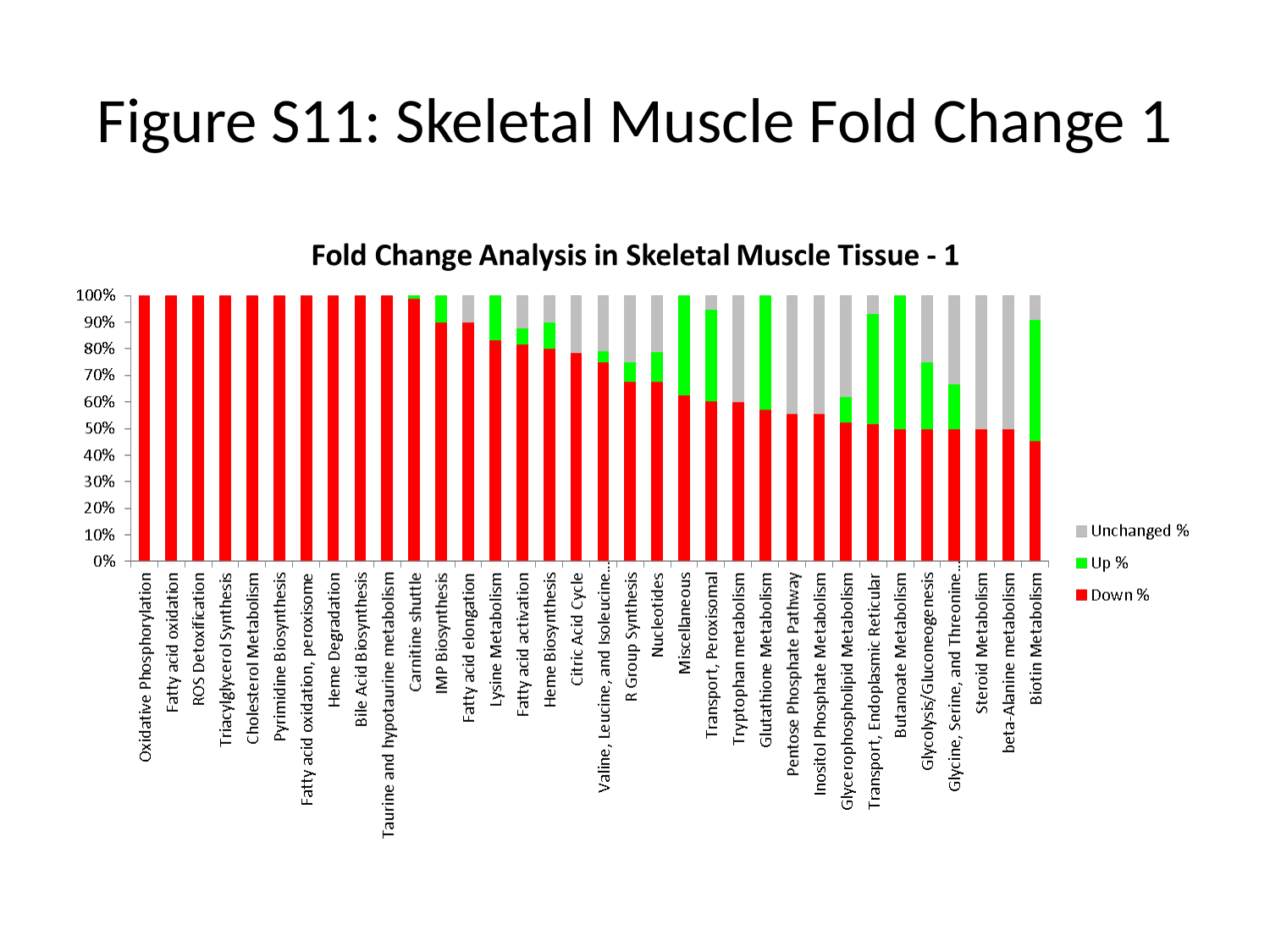

# Figure S11: Skeletal Muscle Fold Change 1

## Slide 12
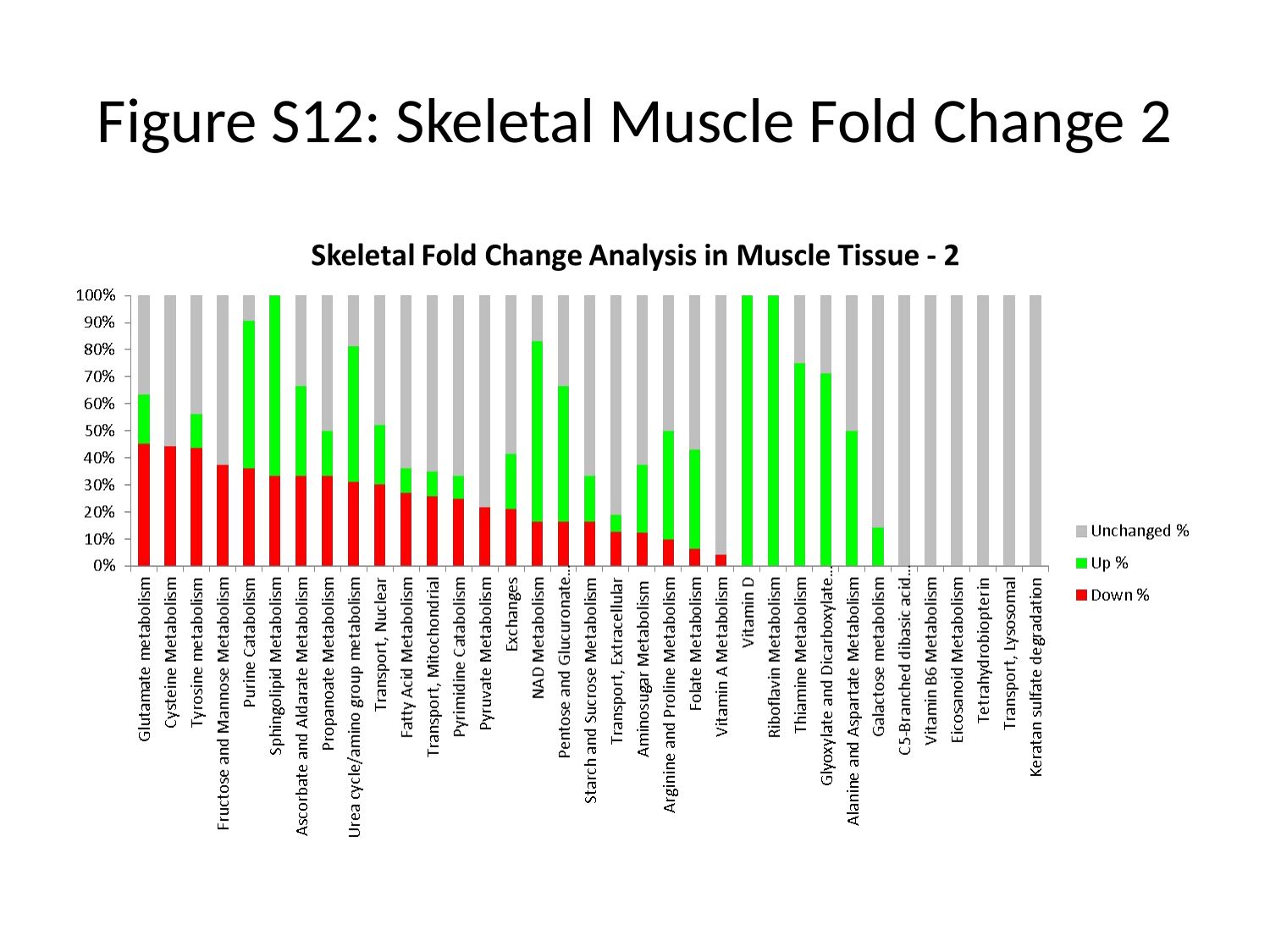

# Figure S12: Skeletal Muscle Fold Change 2

## Slide 13
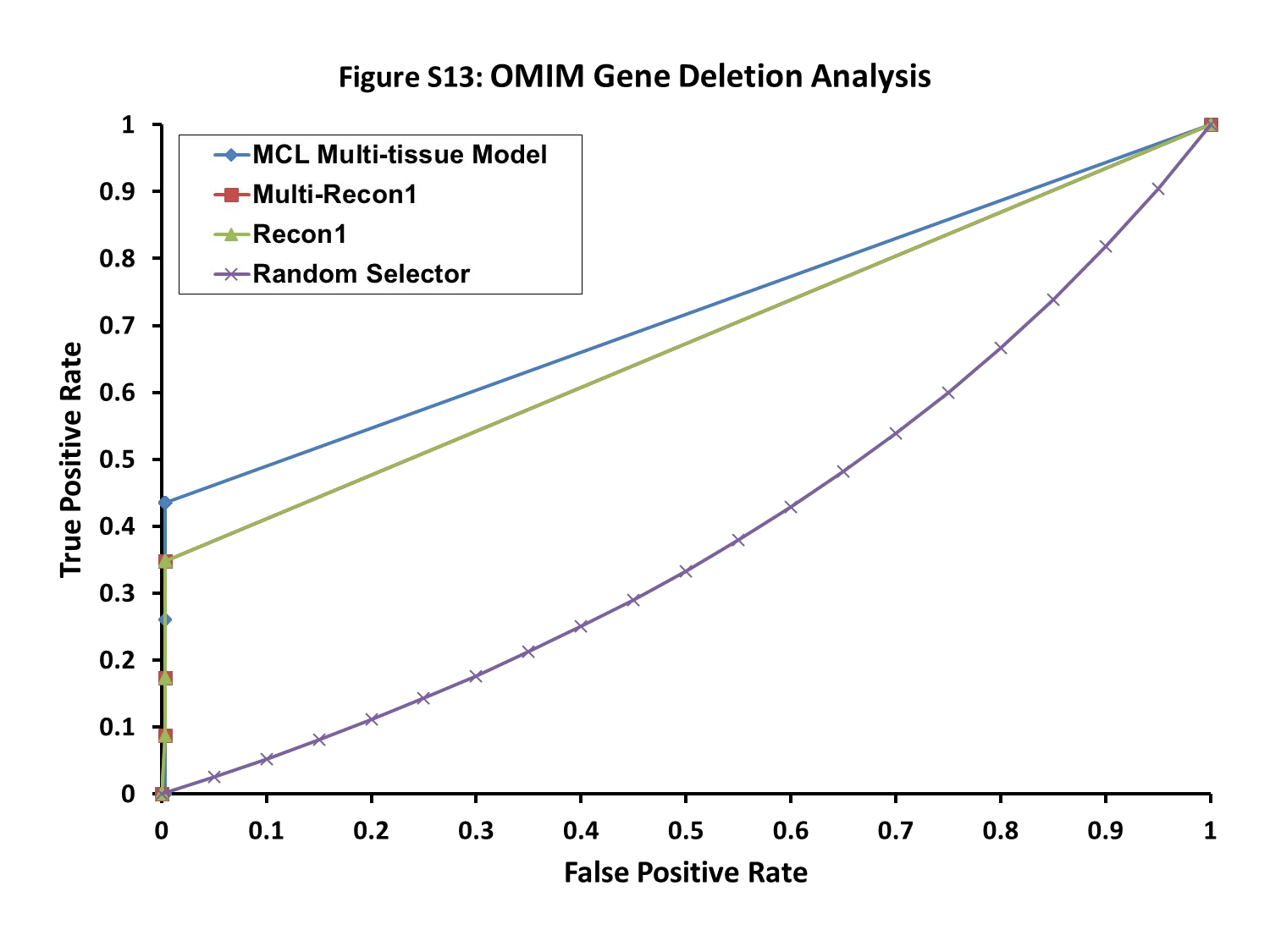

## Slide 14
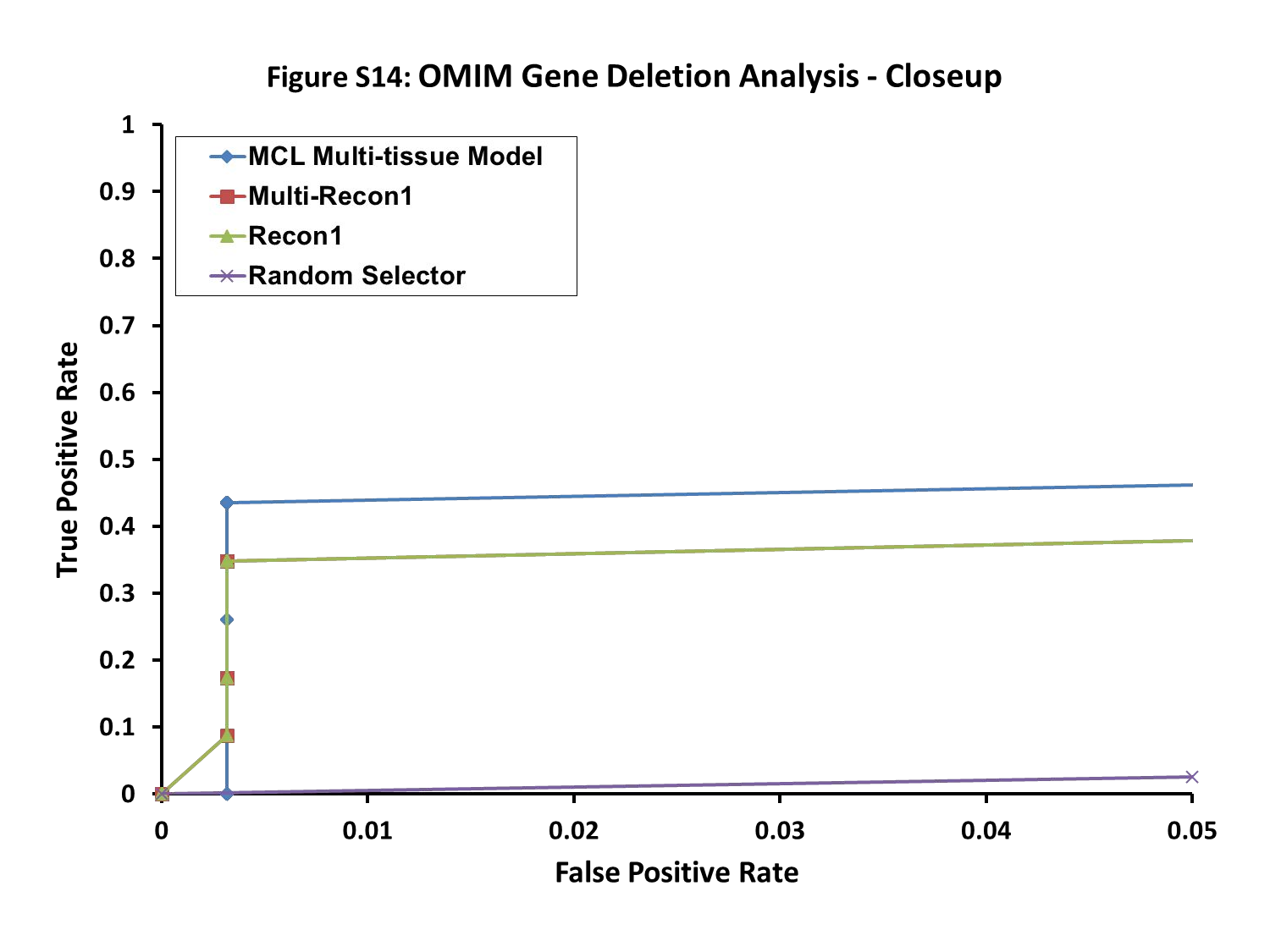

## Slide 15
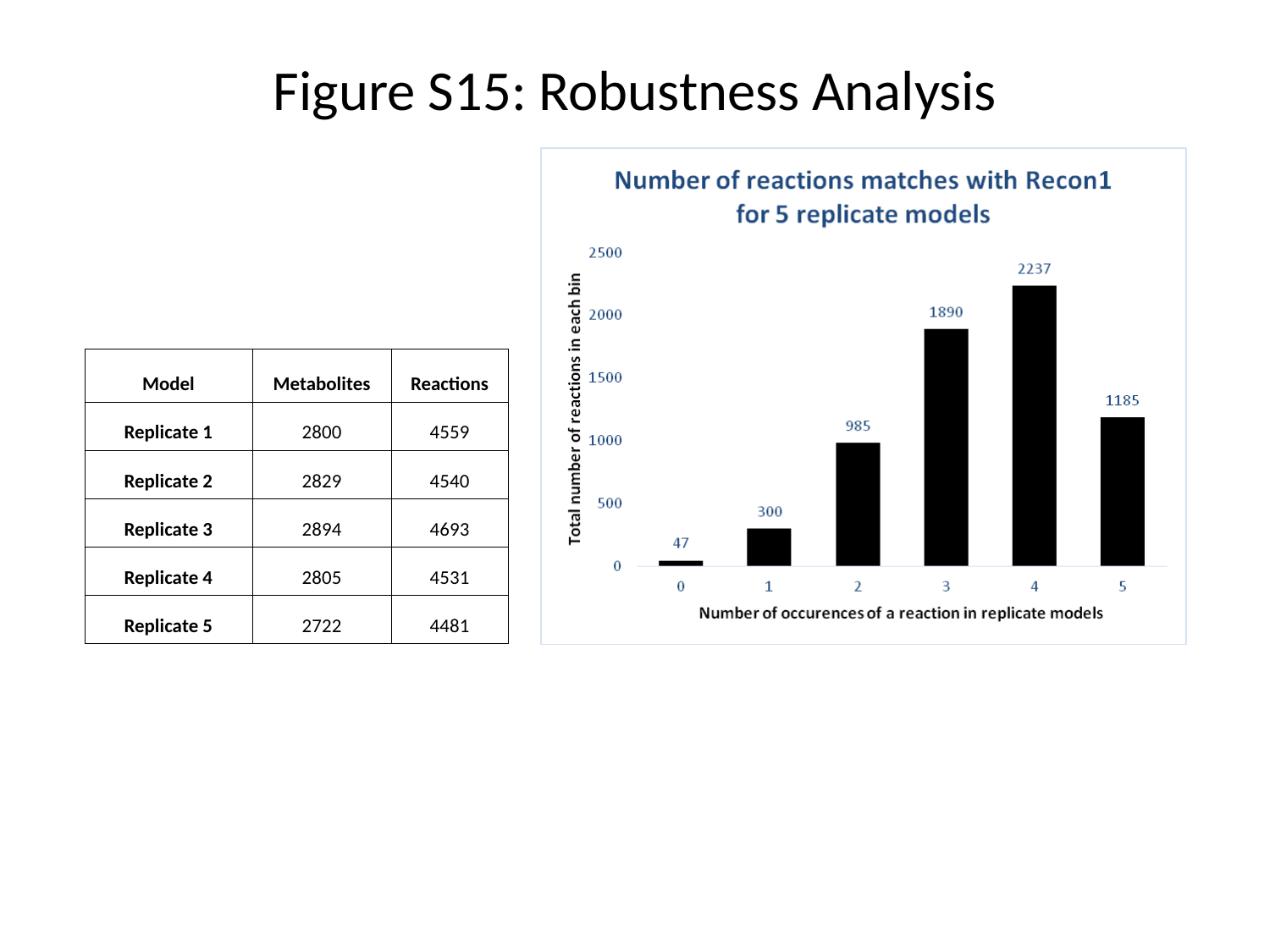

# Figure S15: Robustness Analysis
| Model | Metabolites | Reactions |
| --- | --- | --- |
| Replicate 1 | 2800 | 4559 |
| Replicate 2 | 2829 | 4540 |
| Replicate 3 | 2894 | 4693 |
| Replicate 4 | 2805 | 4531 |
| Replicate 5 | 2722 | 4481 |
